# An air-stable Dy(iii) single-ion magnet with high anisotropy barrier and blocking temperature†
†Electronic supplementary information (ESI) available: Synthetic details, crystallographic details, magnetic studies and quantum mechanical calculations. CCDC 1429571–1429573. For ESI and crystallographic data in CIF or other electronic format see DOI: 10.1039/c6sc00279j


**DOI:** 10.1039/c6sc00279j

**Published:** 2016-04-13

**Authors:** Sandeep K. Gupta, Thayalan Rajeshkumar, Gopalan Rajaraman, Ramaswamy Murugavel

**Affiliations:** a Department of Chemistry , Indian Institute of Technology Bombay , Mumbai-400076 , India . Email: rmv@chem.iitb.ac.in

## Abstract

Herein we report air-stable Dy(iii) and Er(iii) single-ion magnets (SIMs) with pseudo-*D*_5h_ symmetry, synthesized from a sterically encumbered phosphonamide, ^*t*^BuPO(NH^i^Pr)_2_, where the Dy(iii)-SIM exhibits a magnetization blocking (*T*_B_) up to 12 K, defined from the maxima of the zero-field cooled magnetization curve, with an anisotropy barrier (*U*_eff_) as high as 735.4 K. The Dy(iii)-SIM exhibits a magnetic hysteresis up to 12 K (30 K) with a large coercivity of ∼0.9 T (∼1.5 T) at a sweep rate of ∼0.0018 T s^–1^ (0.02 T s^–1^). These high values combined with persistent stability under ambient conditions, render this system as one of the best-characterized SIMs. *Ab initio* calculations have been used to establish the connection between the higher-order symmetry of the molecule and the quenching of quantum tunnelling of magnetization (QTM) effects. The relaxation of magnetization is observed *via* the second excited Kramers doublet owing to pseudo-high-order symmetry, which quenches the QTM. This study highlights fine-tuning of symmetry around the lanthanide ion to obtain new-generation SIMs and offers further scope for pushing the limits of *U*_eff_ and *T*_B_ using this approach.

## Abstract 2

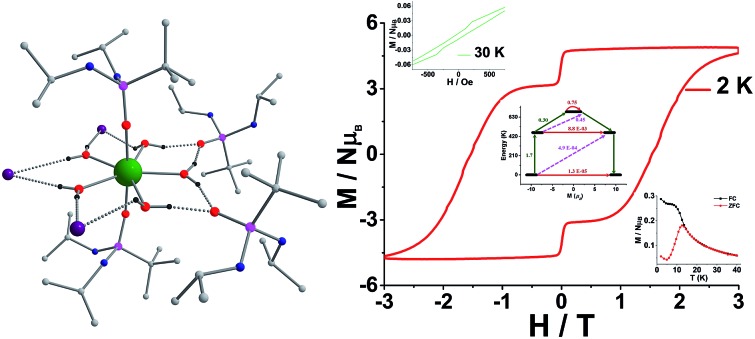
A mononuclear Dy(iii) complex assembled just from five water molecules and two phosphonic diamide ligands combines the advantages of high anisotropy barrier, high blocking temperature and significant coercivity, apart from its remarkable air- and moisture-stability.

## Introduction

Single-ion magnets (SIMs), that exhibit magnetization blocking below a critical temperature (*T*_B_), represent the ultimate size limit for future spin-based devices. Raising *T*_B_ is an ongoing unresolved challenge and lanthanide ions are found to be the most appealing candidates.^1^–^3^ Further for futuristic technological applications/fabrications,^4^ the single molecule magnets (SMMs) must have high anisotropy barrier (*U*_eff_) for reversal of magnetization with a significant blocking temperature (*T*_B_), coercivity and ambient stability.

A very large barrier for a mononuclear lanthanide complex was reported originally for (Bu_4_N)[Tb(Pc)_2_] by Ishikawa *et al.*^5^ Later on such behaviour was also observed for polynuclear lanthanide systems such as {Dy_4_K_2_}, {Dy_5_} and {Dy_3_} triangles.1a,^6^ Combination of lanthanide ion with either 3d metal ions or radicals as ligands, add another dimension to this area where combination of Tb(iii) with the N_2_^3–^ radical led to the isolation of {[(Me_3_Si)_2_N]_2_(THF)Ln]_2_(μ-η^2^:η^2^-N_2_)}^–^ SMM possessing a record blocking temperature of 14 K observed for any SMMs.1*c* The mechanism of magnetic relaxation observed in lanthanide-based molecular magnets are distinctly different to those of the transition-metal ion based SMMs. Particularly the large Spin–Orbit (SO) coupling associated with the lanthanide ions not only yield large magnetic anisotropy, but also allow different *m*_*J*_ levels to mix to a certain extent, leading to QTM between the ground or first excited Kramers doublet (KD). Due to these reasons, *U*_eff_ in lanthanide complexes is not always translated to *T*_B_. This is evident from the fact that *U*_eff_ as large as 938 K been reported but the blocking temperature is still only a fraction of this number.1*d*

Several strategies have been employed to quench the QTM process in lanthanide-based molecular magnets. Amongst these, inducing a strong exchange coupling in the cluster has been found to quench the QTM effects.1b,c As lanthanide ions exhibit only a weak magnetic exchange, generally this is done by incorporating a radical or a 3d metal ion in the cluster aggregation. Earlier significant examples of this category are: (a) N_2_^3–^ radical-based lanthanide complexes that exhibit very large exchange interaction and (b) a {Cr_2_Dy_2_} complex exhibiting hysteresis up to 2.2 K.1b,c,^7^ Despite these efforts, enhancing the exchange interaction significantly to quench the QTM of the ground and other excited states is still a challenge, as the 4f orbital can only feebly interact with the localized radical centres. An alternative strategy to achieve this behaviour is to control the coordination geometry and hence the symmetry around the lanthanide ions to quench the prominent deactivating QTM through appropriately designed molecules with higher order symmetry.^8^ Recently, trimetallic {3d-Dy} systems, M(ii)–Dy(iii)–M(ii) possessing either high symmetry (quasi-*D*_5h_) or low symmetry around Dy(iii) have been reported to exhibit higher barrier heights, however with rather low coercivity values.^9^ If high-order symmetry is preserved, these forbid the mixing of wave functions and hence quench the QTM effects. In this regard, low-coordinate lanthanide complexes are attractive. Generally, synthesis of low-coordinate transition-metal and lanthanide complexes is extremely challenging as it not only requires inert conditions, but also the complexes made *via* this route are generally unstable under ambient aerobic conditions.3b,^10^

To obviate the aforementioned difficulties in stabilizing systems with higher symmetry, large *T*_B_, *U*_eff_ and significant coercivity, we have attentively designed and synthesized novel air-stable Dy(iii) and Er(iii) complexes of phosphonic diamide, which possess a pseudo-*D*_5h_ symmetry. The Dy(iii) SIM exhibits a magnetization blocking (*T*_B_) up to 12 K with an anisotropy barrier (*U*_eff_) as high as 735.4 K and magnetic hysteresis up to 12 K (30 K) with a large coercivity ∼0.9 T (∼1.5 T) at a field sweep rate of ∼0.0018 T s^–1^ (∼0.02 T s^–1^). These high values combined with ambient stability makes the Dy(iii) SIM as one of the best SIMs. Further *ab initio* calculations have been performed to understand the role of the phosphonamide ligand and the higher order symmetry around Dy(iii) ion which result in the realization of such novel properties.

## Results and discussion

### Synthetic aspects

**Scheme 1:**
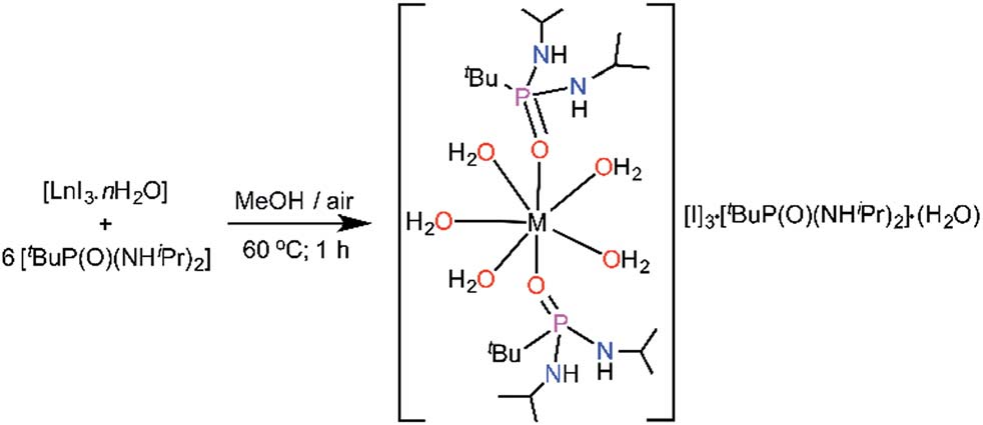
Synthesis of the seven-coordinate complexes **1–3**.

The monometallic seven-coordinate Dy(iii) and Er(iii) complexes of the phosphonic diamide ligand,^11^^*t*^BuPO(NH^i^Pr)_2_ (L) were synthesised as shown in Scheme 1. The presence of sterically bulky alkyl groups in the phosphonamide ligand are highly desirable to maintain larger intermetallic distances in the lattice and thereby reducing the intermolecular interactions. Reaction of the metal iodide hydrate and the phosphonic diamide in 1 : 6 molar ratio in methanol followed by crystallisation under ambient conditions led to the isolation of novel air-stable Dy(iii) and Er(iii) complexes, [L_2_Ln(H_2_O)_5_][I]_3_·L_2_·(H_2_O) [Ln = Dy (**1**); Er (**2**); L = (^*t*^BuPO(NH^i^Pr)_2_)]. Further to carry out the dilution experiments we have synthesised the isomorphous yttrium analogue (**3**). All the compounds have been characterized by FTIR, elemental analysis and the yttrium analogue have further been characterized by NMR spectroscopy (see ESI†).

### Molecular structures

**Fig. 1:**
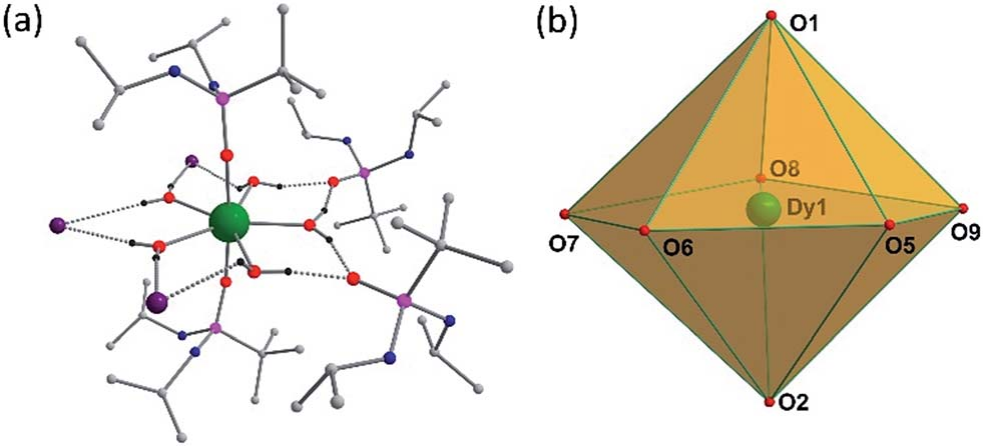
(a) Molecular structure of **1**. Lattice water molecule and most of the H-atoms have been omitted for clarity. The H-atoms of the water molecules are hydrogen bonded to the three iodide anions and two lattice phosphonic diamide ligands. (b) Polyhedron showing *D*_5h_ symmetry around Dy^III^ ion.

Solid-state molecular structures of **1–3** were determined by single-crystal X-ray diffraction studies. Single-crystal X-ray structure analysis of a block shaped crystal reveals that **1** crystallizes in the centrosymmetric triclinic space group *P*1[combining macron]. The asymmetric part of the unit cell contains a single Dy(iii) ion in a pseudo-*D*_5h_ symmetry with the five equatorial coordination sites being occupied by water molecules and the two axial coordination sites being occupied by the phosphonic diamide ligands coordinated to the metal through the phosphoryl oxygen (P=O) (Fig. 1).

The distortions from the ideal seven-coordinate geometries for the {DyO_7_} core are computed using SHAPE software^12^ and the least deviation has been found for the pentagonal bipyramidal geometry, suggesting presence of pseudo-*D*_5h_ symmetry for the core geometry (Table S5†). The axial Dy–O distances (Dy–O1(P) 2.208(2), Dy–O2(P) 2.203(2) Å) are significantly shorter than the five equatorial Dy–O(aqua) distances (2.355(3) to 2.375(3) Å), indicating stronger coordination by the phosphonic diamide ligands. It is instructive to note that the *trans* O1–Dy–O2 angle of 175.14(9)° along the axial direction and the equatorial O–Dy–O angles of 70.43(9)–73.52(10)° (sum = 360.31°) clearly support the metal ion exhibiting a pseudo-*D*_5h_ geometry (Table S2†). The shortest Dy···Dy distance in the lattice is 10.819 Å, this large value being largely aided by the presence of two uncoordinated lattice phosphonamide ligands, one lattice water and three iodide ions per formula unit. The isomorphous Er(iii) (**2**) and Y(iii) (**3**) display similar structural features (Table S3 and S4†).

### Magnetic studies

**Fig. 2:**
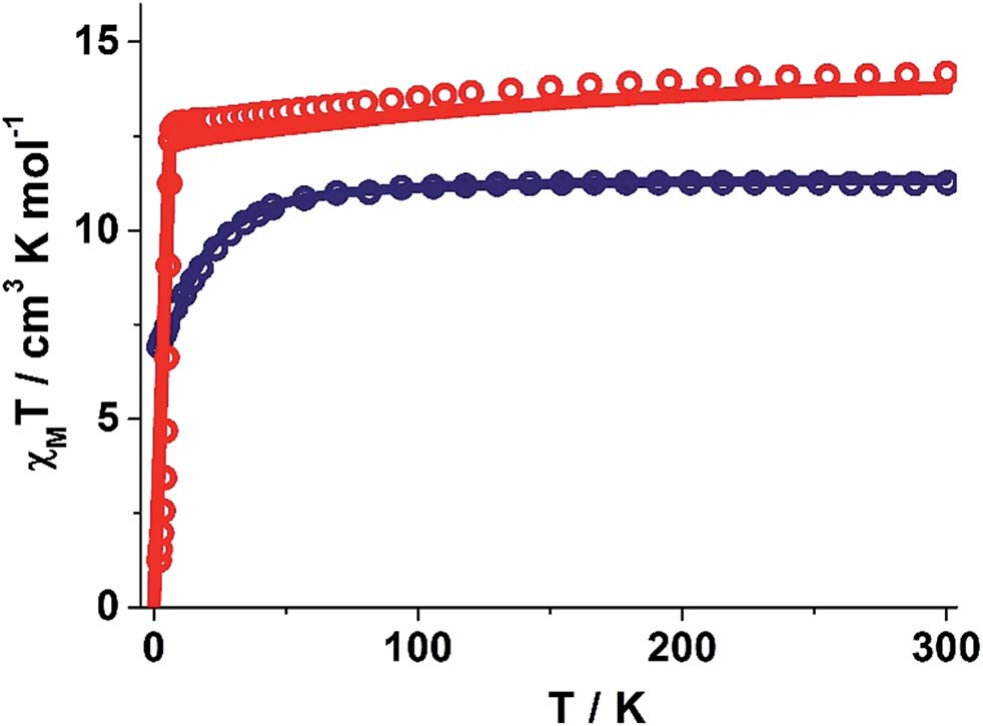
Experimental and *ab inito* CASSCF computed temperature dependence of the *χ*_M_*T* product at 1000 Oe for **1** and **2**. Red and blue hollow circles correspond to the experimental magnetic susceptibility data for **1** and **2**. The solid lines are the computed magnetic susceptibilities. The intermolecular interaction is assumed to be –0.02 cm^–1^ in the calculations.

The static and dynamic magnetic susceptibility measurements of **1** and **2** have been carried out on powder polycrystalline samples using a MPMS-XL SQUID magnetometer equipped with a 7 T magnet. The direct current (dc) susceptibility measurements of **1** carried out in the temperature range 2–300 K in an applied field of 0.1 T shows *χ*_M_*T* value of 14.15 cm^3^ K mol^–1^ at 300 K (Fig. 2), which is very close to the expected value of 14.18 cm^3^ K mol^–1^ for an isolated Dy^III^ ion (ground state = ^6^H_15/2_). On cooling, the *χ*_M_*T* value gradually decreases to a value of 12.80 cm^3^ K mol^–1^ before steeply decreasing to 1.24 cm^3^ K mol^–1^ at 2.0 K. Complex **2** also shows a similar behavior with a *χ*_M_*T* value of 11.26 cm^3^ K mol^–1^ at 300 K (Fig. 2) which is again close to the expected value of 11.48 cm^3^ K mol^–1^ for an isolated Er^III^ ion (ground state = ^4^I_15/2_). Cooling the sample below 300 K shows a gradual decrease in *χ*_M_*T* value to 11.0 cm^3^ K mol^–1^ at ∼69 K; further cooling results in a sharp decrease in *χ*_M_*T* value to 6.90 cm^3^ K mol^–1^ at 2.0 K. The field (*H*) dependent magnetization (*M*) curve for **1** shows a steep increase (sinusoidal behaviour) in magnetization at lower field (Fig. S5†) before reaching 5.03 *μ*_B_ at 7.0 T. The sigmoidal nature of the magnetization plot at lower fields is anticipated in systems which are highly anisotropic and are generally due to the interactions of the highly anisotropic ions, as usually observed in metamagnets evidenced in earlier literature reports.3g,^13^ Contrary to **1**, the *M vs. H* plot for **2** shows a steep increase in one single step at lower field up to 1.0 T (Fig. S6†). Further increase shows a gradual increase in magnetization up to 7 T at 4.88 *μ*_B_.

**Fig. 3:**
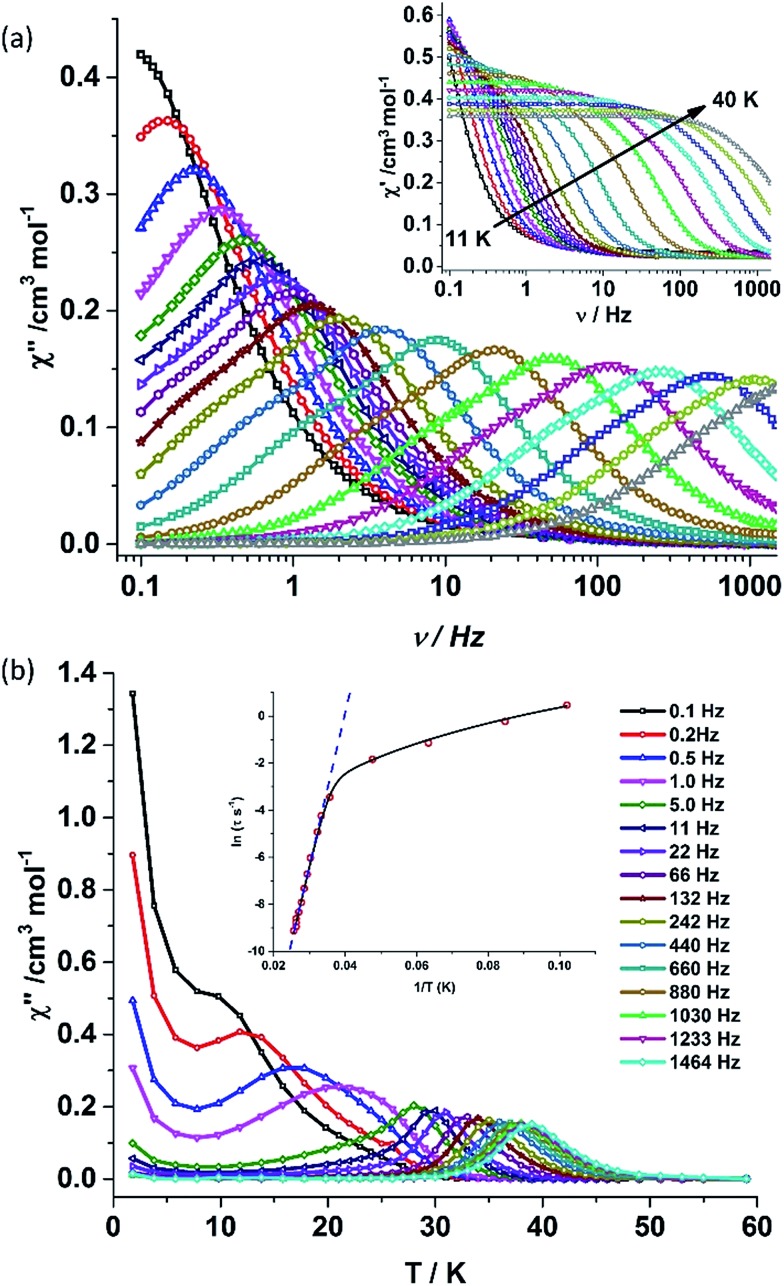
(a) Out-of-phase (*χ*_M_′′) component (inset: in-phase component) of the frequency dependent (0.1–1464 Hz) ac susceptibility measured in the temperature range of 11–40 K in an oscillating ac field of 3.5 Oe and zero applied dc field for **1**. (b) Out-of-phase (*χ*_M_′′) component of the temperature dependent ac susceptibility in an oscillating ac field of 3.5 Oe and zero applied dc field for **1**. (Inset) Plot of the relaxation time *τ* (logarithmic scale) *versus T*^–1^ obtained; the dashed blue line corresponds to the fitting of the Orbach relaxation process and the solid black line represents the best fitting to the multiple relaxation process for **1**.

Alternating current (ac) susceptibility measurements were carried out to unravel the slow relaxation dynamics of magnetization of **1** and **2**. AC susceptibility measurements carried out on **1** at zero applied dc field between 0.1 and 1464 Hz at an oscillating ac field of 3.5 Oe shows a frequency dependent maxima in the out-of-phase susceptibility component *χ*_M_′′ at higher temperatures indicating a very high thermal energy barrier (Fig. 3(a)). Best fitting of the magnetic relaxation time (*τ*) with the Arrhenius equation *τ* = *τ*_0_ exp(*U*_eff_/*k*_B_*T*) (Fig. 3(b): inset) results in a large energy barrier of *U*_eff_ = 651.0 K and a pre-exponential factor (*τ*_0_) = 5.63 × 10^–12^ s, indicating a very slow relaxation of magnetization.‡
‡The value of *U*_eff_ varies only marginally when its value is computed from the frequency dependent data. See ESI for more information. However below 30 K, the fit to the Arrhenius law deviates from linearity indicating the presence of other possible relaxation pathways (Fig. 3(b)).3b,e,^14^ The fitting of the Cole–Cole plots (*χ*_M_′′ *vs. χ*_M_′) with the Debye model considering two relaxation processes indicate the presence of a narrow distribution of relaxation time (0.076 < *α*_1_ < 0.111 and 0 < *α*_2_ < 0.110) (Fig. S8†). It has been previously ascribed in the literature that the relaxation in the range 12–30 K cannot be attributed to QTM.3*g* Fitting of the relaxation time using multiple relaxation processes indicates that the relaxation occurs *via* the temperature dependent Orbach and Raman mechanism (Fig. S9 and S10†).

**Fig. 4:**
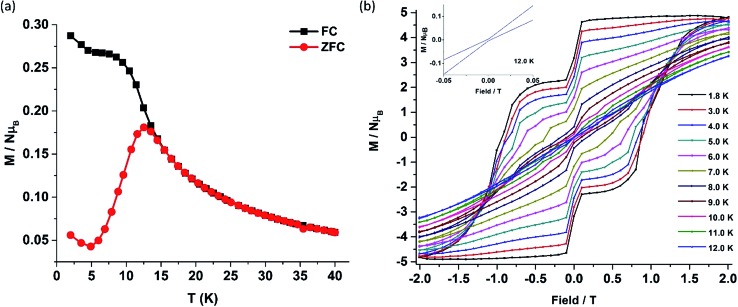
(a) Plot of zero field-cooled (red) and field-cooled (black) magnetization *vs.* temperature for **1**. (b) The field-dependent magnetization data for **1** were collected at a sweep rate of 0.0018 T s^–1^ sweeping the field from +2 T to –2 T and back to +2 T in the temperature range 1.8–12.0 K. (Inset; expansion of the *M–H* curve at 12.0 K.)

The deviation of the variable-temperature zero field-cooled (ZFC) magnetization data from the field-cooled (FC) data with the maximum in ZFC curve at 12 K (*T*_B_) indicates blocking of the molecular spin (Fig. 4(a)), as defined by Gatteschi *et al*.1*g* In order to further investigate the SIM behaviour in **1** corresponding to the blocking of magnetization, field dependent magnetization measurements were carried out at different temperatures at an average sweep rate of 0.0018 T s^–1^ (time for full cycle). The field was swept from 0 T to +2 T and then to –2 T and back. A wide butterfly shaped hysteresis loop was observed at 1.8 K with a large coercive field of *H*_c_ about ∼0.9 T (Fig. 4(b)). The loops remain wide open until 10 K (*H*_c_ about ∼500 Oe) before slowly approaching a value *H*_c_ ∼ 0.0 T at 12.5 K (Fig. S11†). As opening of the hysteresis loop is highly dependent on field sweep rate and the method of measurement (Table 1), a further increase in the sweep rate to 0.02 T s^–1^ using continuous sweep mode leads to opening of the hysteresis loop at least up to 30 K with a large coercivity of ∼1.5 T at 2 K (Fig. S12†).

**Table 1:** Comparison of Dy(iii)-SIM with some mononuclear 4f, polynuclear 4f and 3d–4f-based SMMs^*d*^

Complex	*U*_eff_/K	ZFC maxima (*T*_B_)/K	Hysteresis		Coercivity/*T*	Ambient air stability	Ref.
			*T*/K	T s^–1^			
**1**	651.0, 735.4^*c*^	12	12^*a*^	0.0018	∼0.9 (1.8 K)	Yes	This work
			30^*b*^	0.02	∼1.5 (2.0 K)		
[Dy(BIPM^TMS^)_2_][K(18C6)(THF)_2_]	721, 813	10	16	0.0035	<0.7 (1.8 K)	No	3*g*
					<0.8 (1.8 K)^*c*^		
(Cp*)Er(COT)	323, 197	5^*c*^	5.0	0.000916	∼0 (1.8 K)	No	2*a*
					∼0.01 (1.8 K)^*c*^		
(Cp*)Er(COT)	323, 197	—	—	0.07^*c*^	1.3 (1.6 K)^*c*^	No	2*b*
Er[N(SiMe_3_)_2_]_3_	122	—	1.9	—	∼0	No	10*d*
[Li(DME)_3_][Er^III^(COT′′)_2_]	187	—	8.0	0.0022	0.6250 (1.8 K)	No	1*f*
[Er(COT)_2_]^–^	286	—	12	0.0035	0.7 (1.8 K)	No	3*a*
[Er(COT)_2_]^–^	211.5	10^*c*^	10	0.00078	0.7 (1.8)	No	15
					1.1 (1.8 K)^*c*^		
[Li(THF)_4_[Er{N(SiMe_3_)_2_}_3_Cl]·2THF	63.3	—	3^*c*^	0.00346	<0.02 (1.8 K)^*c*^	No	3*b*
[Pc_2_Tb]TBA	331	—	—	—	—	No	5
[TbPc_2_]/[TBA][Br]	922^*c*^	—	—	—	—	No	16
[TBA][Tb{Pc(phth^3^)}_2_]	666.2		2.0	0.01666	<0.03 (2.0 K)	No	17
[TbPc_2_]	590	—	—	—	—	No	18
[Tb{Pc(OEt)_8_}_2_][SbCl_6_]	791	—	—	—	—	No	19
[TBA][Tb{Pc(OEt)_8_}_2_]	732	—	—	—	—	No	19
[Tb{Pc(S-DOP)_8_}_2_]	690	—	—	—	—	No	20
[Tb(Pc)(Pc′)]^–^	938	—	—	—	—	No	1*d*
[(Cp′_2_Dy){μ-P(H)Mes}]_3_	302, 368^*c*^	—	4.4^*c*^	0.0026^*c*^	∼0.03 (1.8 K)^*c*^	No	21
[K(18C6)]{[(Me_3_Si)_2_N]_2_(THF)Dy}_2_(μ-η^2^:η^2^-N_2_)	177	8.3	8.3	0.08	∼1.5 (2–6 K)	No	1*b*
[Dy_4_K_2_O(O^*t*^Bu)_12_]·C_6_H_14_	692, 316	—	5.0	0.14	<0.15 (0.03 K)	No	1*a*
	842^*c*^		6.0^*c*^		∼0.25 (0.03 K)^*c*^		
[Dy_5_(μ_5_-O)(μ_3_-O^i^Pr)_4_(μ-O^i^Pr)_4_(O^i^Pr)_5_]	528	—	1.85	—	—	No	1*a*, 6*a*
	804^*c*^		7^*c*^	0.001^*c*^	∼0^*c*^		
[[K(18C6)(THF)_2_][{[(Me_3_Si)_2_N]_2_(THF)Tb}_2_(μ-η^2^:η^2^-N_2_)]	326	14	14	0.0009	<5.0 (11 K)	No	1*c*
[(η^5^-Cp)_2_Dy(μ-bpym)]_2_[BPh_4_]	127	6.5	6.5	0.002	∼0.6 (3 K)	No	2*d*
[Dy(hfac)_3_(μ-pyNO)]_2_	167	—	1.4	0.02	∼0.0121 (1.4 K)	Yes	22
{[Cp′_2_Dy(μ-SSiPh_3_)]_2_	192	—	1.8	—	∼0	No	23
[ErIII2(COT′′)_3_]	323	—	12	0.0022	<0.2 (1.8 K)	No	13*a*
K_2_(THF)_4_[ErIII2(COT)_4_]	306	—	12	0.0018	∼0 (1.8 K)	No	13*a*
[Zn_2_(L^1^)_2_DyCl_3_]·2H_2_O	430	<4.5	8	0.000166–0.0005	∼0 (1.8 K)	Yes	9*b*
			12	0.02	—		
	434^*c*^	—	—	—	0.03 (1.8 K)^*c*^		
[Zn_2_(L^1^)_2_Dy(MeOH)Br_3_]·3H_2_O	233	<3.5	6	0.000166–0.0005	∼0 (1.8 K)	Yes	9*b*
[Zn_2_(L^1^)_2_Dy(H_2_O)Br_2_]·[ZnBr_4_]_0.5_	121	<2.5	4	0.000166–0.0005	∼0 (1.8 K)	Yes	9*b*
[Zn_2_(L^2^)_2_DyCl_3_]·2H_2_O	398	<4.5	8	0.000166–0.0005	∼0 (1.8 K)	Yes	9*b*
[Fe_2_Dy(L^3^)_2_(H_2_O)]ClO_4_·2H_2_O	459	—	—	—	—	Yes	9*c*
[Zn_2_Dy(L^4^)_2_(MeOH)]NO_3_	439	—	11	0.02	>0.02 (2.0 K)	Yes	9*d*
[Co_2_Dy(L^4^)_2_(H_2_O)]NO_3_	600	—	—	—	—	Yes	9*a*

^*a*^Hysteresis mode.

^*b*^Continuous sweep mode.

^*c*^Diluted in diamagnetic matrix.

^*d*^(—) = not reported. (∼) = close to but not equal to. BIPM^TMS^ = {C(PPh_2_NSiMe_3_)_2_}^2–^; 18C6 = 18-crown-6; Cp* = pentamethylcyclopentadienide; COT = cyclooctatetraene; DME = dimethoxyethane; COT′′ = 1,4-bis(trimethylsilyl)cyclooctatetraenyl dianion; THF = tetrahydrofuran; Pc = dianion of phthalocyanine; TBA = tetra-*n*-butylammonium; Pc(phth^3^) = [bis(*N*,*N*,*N*,*N*-tetra((*S*)-methyl(phenyl)methyl)-29*H*,31*H*-2,3,9,10,16,17,23,24-phthalocyaninatotetradicarboximide); Pc(OEt)_8_ = dianion of 2,3,9,10,16,17,23,24-octaethoxyphthalocyanine; SDOP = (*S*)-2-(dodecyloxy)propan-1-oxy; Pc′ = octa(tert-butylphenoxy)-phthalocyanine; Cp′ = η^5^-C_5_H_4_Me; Mes = mesityl; bpym = 2,2′-bipyrimidine; hfac = hexafluoroacetylacetonate; PyNO = pyridine-*N*-oxide; H_2_L^1^ = *N*,*N*′-bis(3-methoxysalicylidene)phenylene-1,2-diamine; H_2_L^2^ = *N*,*N*′-bis(3-methoxysalicylidene)-1,2-diaminocyclohexane; L^3^ = 2,2′,2′′-(((nitrilotris(ethane-2,1-diyl))tris(azanediyl))tris(methylene))tris-(4-chlorophenol); L^4^ = 2,2′,2′′-(((nitrilotris(ethane-2,1-diyl))tris(azanediyl))tris(methylene))tris-(4-bromophenol).

**Fig. 5:**
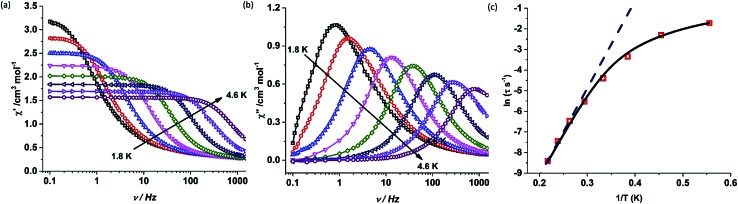
(a) In-phase (*χ*_M_′) and (b) out-of-phase (*χ*_M_′′) component of the ac susceptibility measured in the frequency range of 0.1–1464 Hz in an oscillating ac field of 3.5 Oe under an applied dc field of 2000 Oe for complex **2**. (c) Plot of the relaxation time *τ* (logarithmic scale) *versus T*^–1^; the dashed blue line corresponds to fitting of the Orbach relaxation process and the solid black line represents the best fitting to the multiple relaxation process.

Contrary to complex **1**, complex **2** shows a drastic change in magnetic properties and does not exhibit any maxima in the out-of phase (*χ*′′) ac susceptibility signals under zero applied dc field (Fig. S14†). However, the application of an optimum dc field of 2000 Oe (Fig. S15†) resulted in well-resolved out-of-phase ac susceptibility maxima (Fig. 5 and S17†). The energy barrier for the thermal relaxation in **2** was extracted using the Arrhenius relationship which gave *U*_eff_ = 44.7 K, with a pre-exponential factor of *τ*_0_ = 1.08 × 10^–8^ s.‡ In the case of **1**, the application of a dc field of 2000 Oe increases the barrier height to *U*_eff_ = 705.3 K, with a pre-exponential factor of *τ*_0_ = 5.83 × 10^–12^ s (Fig. S21–S23†) indicating the presence of tunnelling either due to hyperfine or intermolecular interactions.3h,^24^ It is observed that on the application of dc field, the broadening of the peaks at lower frequency is diminished and shifts to higher temperatures. However the fit to the Arrhenius law deviates from linearity below 31 K (Fig. S23†).

**Fig. 6:**
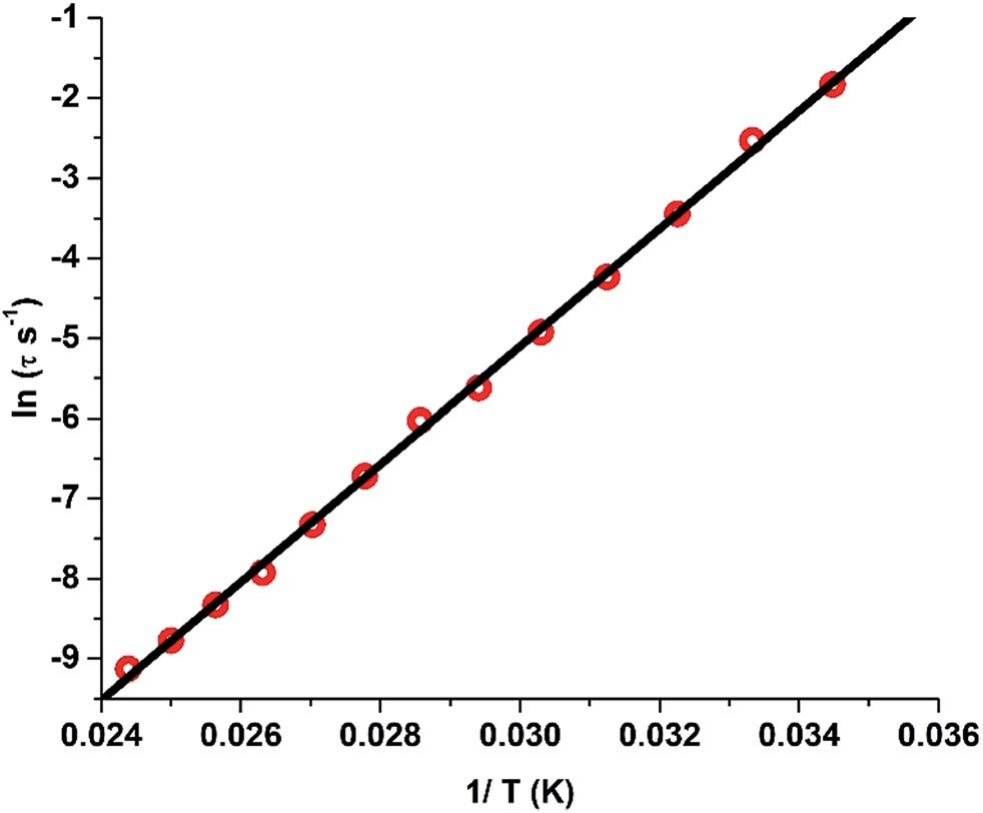
Plot of the relaxation time *τ* (logarithmic scale) *versus T*^–1^ for **1′**; the solid red line corresponds to fitting of the Orbach relaxation process.

Further dilution experiments have been carried out to understand the influence of intermolecular interactions on the magnetic properties of **1**. In this regard, the isomorphous yttrium analogue **3** has been prepared and characterized, which has been employed to generate the diluted sample, [L_2_Dy_0.25_Y_0.75_(H_2_O)_5_][I]_3_·L_2_·(H_2_O)] (**1′**) (prepared by accurately weighing analytically pure crystalline samples of **1** and **3** in 1 : 3 molar ratio and dissolving in a minimum amount of dichloromethane and benzene). The clear solution obtained was vigorously stirred for 1 h and the solvent was slowly allowed to evaporate to obtain a crystalline powder. SQUID response data have been obtained for this sample, whose dynamic (ac) magnetic susceptibility properties exhibit a similar behaviour to **1** at higher temperatures while the lower frequency maxima shifts to a higher temperature (Fig. S25†).§
§SQUID response signals were weak for lower levels of dilution (<25%). A fit to the Arrhenius law of the relaxation times leads to an energy barrier of *U*_eff_ = 735.4 K with a pre-exponential time constant of *τ*_0_ = 1.56 × 10^–12^ s (Fig. 6). Contrary to **1**, the Arrhenius plot does not reveal any deviation from linearity at the measured temperatures and frequencies. However, the broadening of the peaks in the lower frequency range indicates the presence of additional relaxation pathways albeit the major relaxation mechanism is primarily the temperature-dependent Orbach and Raman process. The broadening of the peaks at lower frequencies towards lower temperatures could be due to hyperfine interactions.3*h* This also illustrates that the tunnelling observed around zero field in the hysteresis loops is likely to be due to hyperfine interactions.3*h*

### Electronic structure calculations

**Fig. 7:**
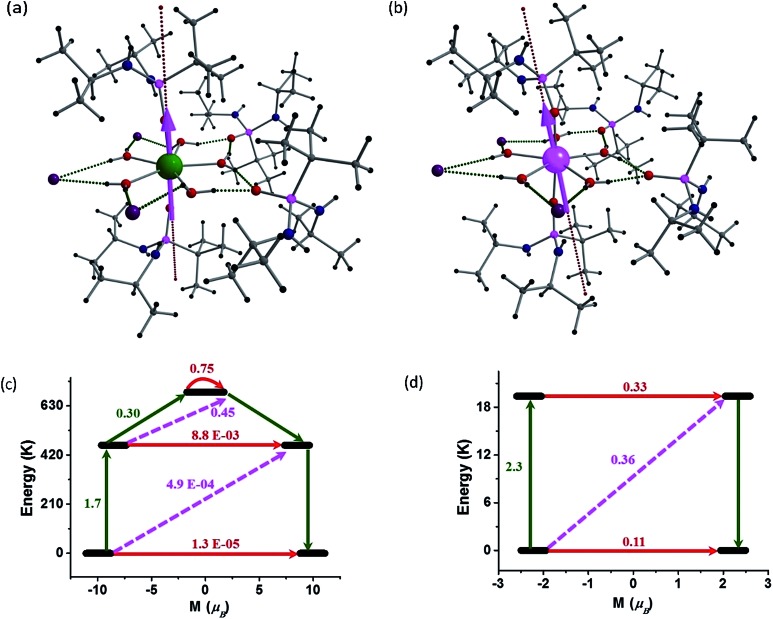
Electronic structures and energy levels for **1** and **2**. (a) and (b) CASSCF computed *g*_*zz*_ orientation of the ground state KD of complexes **1** and **2**. (c) and (d) Possible relaxation pathways in **1** and **2**. The black line indicates the KDs as a function of magnetic moments. Red lines represent QTM *via* ground state, KDs/TA-QTM *via* first and second excited states. Pink dashed lines show possible Orbach process.

In order to understand the contrasting pattern of magnetic relaxation observed for complexes **1** and **2**, we have performed *ab initio* CASSCF/RASSI-SO calculations^8^,^25^ using the MOLCAS 7.8^26^ suite of software. All the calculations have been performed on the X-ray structures of **1** and **2** ([L_2_Ln(H_2_O)_5_][I]_3_·L_2_, excluding water molecules in the crystal lattice) (Fig. 7). Apart from the calculations on the crystal structures, we have also performed calculations on models constructed from crystal structures of **1** and **2** in the following way: model-I (**1a** corresponds to [L′_2_Ln(H_2_O)_5_] and here L′ = (MePO(NHMe)_2_)]), model-II (**1b** corresponds to [L′_2_Ln(H_2_O)_5_][I]_3_·L′_2_) and model-III (**1c** corresponds to ([L_2_Ln]^3+^) where water molecules in equatorial position, counter anions and the co-crystallized ligands are excluded). Earlier we have employed the same theoretical methodology to study a series of Dy(iii) and Er(iii) complexes to assess models which could possibly offer very large barrier heights. We have proposed that the probable ligand interaction (axial for Dy(iii) and equatorial for Er(iii)) accompanied by higher order symmetry should be ideal for attaining higher barrier heights and particularly the importance of high-symmetry around the metal ions to quench the QTM at the ground and excited state was highlighted.25*e* Here the complexes **1** and **2** are real examples coincident with our earlier proposed hypothesis. The computed *g*-tensors of the ground state KDs for **1** is purely Ising in nature (*g*_*xx*_, *g*_*yy*_ = 0.4 × 10^–4^ and *g*_*zz*_ = 19.86) as the *g*_*zz*_ value reaches close to ∼20 and the transverse components are zero. Absence of transverse anisotropy at the ground state suggests quenching of QTM process at this level. The computed eight KDs belonging to the ^6^H_15/2_ ground state of the Dy(iii) ion is found to span up to 1028.4 K. For complex **1**, the ground state is found to be a pure *m*_*J*_ = |±15/2 = |±15/2〉 state (Table S7 state (Table S7†) and the *g*_*zz*_ axis is found to lie nearly along the pseudo-*C*_5_ axis (along the Dy–O–P bond vector with a deviation of 4.3°). The first excited KD is found to lie 461.6 K above the ground state and this state is a pure *m*_*J*_ = |±13/2 = |±13/2〉 state (Table S6 and S7 state (Table S6 and S7†) with a negligible transverse anisotropy (*g*_*zz*_ = 17.08, *g*_*xx*_, *g*_*yy*_ = 0.02). This shows that the KDs of the first excited state are strongly axial in nature and emphasizes the quenching of QTM even at this level. The *g*_*zz*_ orientation of the first excited state KD is also found to orient along the pseudo-*C*_5_ axis. This coincidence of the anisotropic axes suggests (note that the deviation here is ∼6°) that the relaxation is not likely to occur *via* the first excited state. The second excited KD is found to lie at 688.3 K above the ground state and here the *g*-tensor has a significant transverse component (*g*_*zz*_ = 16.53, *g*_*xx*_ = 0.58, *g*_*yy*_ = 3.13). This state is estimated to be an admixture of *m*_*J*_ = |±1/2 = |±1/2〉 and and *m*_*J*_ = |±5/2 = |±5/2〉 states (Table S6 and S7 states (Table S6 and S7†). The *g*_*zz*_ axis is found to be tilted by ∼94° compared to the ground-state KD *g*_*zz*_ orientation. This significant deviation in the angle and the observance of a transverse component at this level suggest that the relaxation is more likely to happen *via* this second excited state. This proposes that the barrier for relaxation is 688.3 K, which is higher than the experimental observations (undiluted sample). The barrier height measured for the undiluted sample is 651 K while it is found to be significantly enhanced upon dilution to 735.4 K, suggesting that the *U*_eff_ values are strongly correlated to the intermolecular interactions as the intermolecular interactions are not taken in to consideration in our calculations. Such behaviour has been noted earlier for some Er(iii) SMMs.25*d*

The calculations on models **1a** and **1b** also confirm the relaxation *via* the second excited state differing mainly in their magnitude of energy barrier (Table S8 and S9†). The second KDs of **1a** and **1b** lie at 1088.9 and 613.3 K, respectively. The difference in the energy barriers of the models can be correlated with the changes in the NPA charges on the oxygen atoms (Table S10†). The NPA charges have been computed to also analyse the influence of charge distributions on the stabilization of *m*_*J*_ levels and the extent of crystal field (CF) splitting. The oxygen atoms of the phosphonic diamide ligands in the axial positions are found to possess significant negative charge, compared to the oxygen atoms of water molecules in **1a**, indicating a scenario predicted earlier by us for {Dy(OH)_2_}^+^.25*e* Inclusion of iodides in the equatorial plane (along with water molecules in **1** and **1b**) tend to stabilize the hydrogen bond and this increases the charges on the oxygen atoms of the coordinated water molecules (O5–O9 in Table S10†), thus resulting in the decrease of energy barrier compared to **1a**. The difference in the energies of the second KDs in **1** and **1b** shows the effects of ligand atoms in the second co-ordination sphere and emphasizes the need to consider these influences while modelling the complexes for calculations. To validate the role played by water molecules in equatorial positions, calculations on **1c** were performed. The results clearly shows the relaxation *via* the fourth excited state pushing the energy barrier to 2980.5 K (Table S11†) and re-iterates our earlier prediction25*e* that two-coordinate geometry is the most favourable co-ordination for Dy(iii) for obtaining large barrier heights.

The computed eight KDs belonging to the ^4^I_15/2_ ground state of the Er(iii) ions is found to span up to 579.7 K (Table S12†). In contrast to complex **1**, the ground state KD of complex **2** shows significant transverse anisotropy [*g*_*xx*_ = 0.13, *g*_*yy*_ = 0.55 and *g*_*zz*_ = 13.34]. This suggests significant QTM being operational for complex **2** at the ground state itself. Here a significant mixing of excited states *m*_*J*_ = |±11/2 = |±11/2〉, , *m*_*J*_ = |±9/2 = |±9/2〉, , *m*_*J*_ = |±7/2 = |±7/2〉 with ground state with ground state *m*_*J*_ = |±15/2 = |±15/2〉 is observed (Table S15 is observed (Table S15†). This further expands the possibility of significant tunnelling at the ground KD level itself. The *g*_*zz*_ axis of the ground state KD is found to be tilted away from the *C*_5_ axis by an angle of 19.34°. The tilt is larger compared to **1** due to the prolate nature of the Er(iii) ion where maximum electron density is expected to lie along the pseudo-*C*_5_ axis. For the same reason, the first excited KD in this case lies at 19.4 K from the ground state KD. As the ligand field is not ideally suited for the Er(iii), the CF splitting observed is smaller than that of the Dy(iii) ion. The NPA calculations (Table S16†) shows the presence of significant negative charge along the axial direction for the prolate Er(iii) ion and this results in a smaller barrier and significant mixing of the *m*_*J*_ levels, as has been experimentally observed in complex **2**.

To obtain further insights into the mechanism of relaxation in both **1** and **2**, transverse magnetic moments that connect the opposite pairs of magnetization have been computed. The probable mechanism of relaxation and the computed energies of the first four KDs are shown in Fig. 7. The negligible transverse magnetic moments between the ground state and the first and second excited state KDs for complex **1** show that the QTM (∼10^–5^*μ*_B_) and TA-QTM (∼10^–3^*μ*_B_) are significantly quenched. On the other hand, TA-QTM *via* second excited state is prominent in complex **1** as discussed earlier based on transverse anisotropy. Besides, the second excited state has a tunnelling probability of 0.45 *μ*_B_, endorsing our earlier statement.

In the case of complex **2**, the QTM between the ground-state doublets is prominent (0.11 *μ*_B_) and offers a probable relaxation pathway. This is in agreement with the observation of no maxima in the out of phase ac signals at zero-field. The first excited state lying at 19.4 K is found to have a significant TA-QTM probability (Fig. 7(d)). Application of dc field quenches the QTM between the ground state KDs to a certain extent, leading to the observation of relaxation *via* the first excited KD. This rationalizes the observed *U*_eff_ for complex **2** in the presence of an applied dc field.

The crystal field parameters are computed (Table S17 and S18†) to elucidate further insights into the mechanism of relaxation, using the following equation as implemented in SINGLE_ANISO code,^27^
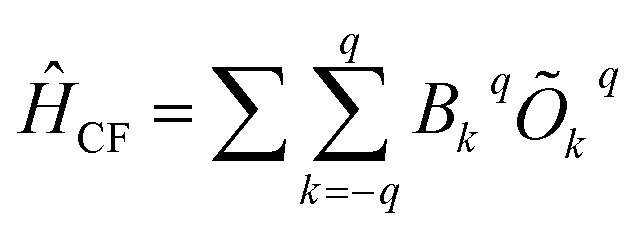
where *Õ*_*k*_^*q*^ and *B*_*k*_^*q*^ are the computed extended Stevens operator and crystal field (CF) parameter, respectively. The probability for the occurrence of QTM is higher when the non-axial *B*_*k*_^*q*^ terms (*q* ≠ 0 and *k* = 2, 4) are larger than the axial (*q* = 0 and *k* = 2, 4) terms. In the case of complex **1**, the axial terms are comparatively larger than the non-axial terms, indicating the absence of QTM. The large negative axial terms compared to non-axial terms stabilizes large *m*_*J*_ value as the ground state. On the other hand, non-axial terms are larger than axial terms inducing QTM between ground-state KD in **2**.

To estimate the strength of intermolecular interaction in complex **1** (the shortest Dy(iii)···Dy(iii) interaction is about ∼10 Å in the crystal lattice), we have fitted the susceptibility data using Lines model^28^ and this yields a weak antiferromagnetic exchange (*J*_inter_ = –0.02 cm^–1^, Fig. 2).

*Note added at proofing*: Since the submission of this work a few interesting Dy(iii)-SIMs with *D*_5h_ symmetry have been reported by Tong and co-workers.^29^

## Conclusions

In summary, mononuclear complex **1** and its 25% diluted sample **1′** have been found to exhibit relaxation barriers as high as 651.0 K (*U*_eff_ = 705.3 K at *H*_dc_ = 2000 Oe) and 735.4 K, respectively. The hysteresis loop of dilute and undiluted samples of complex **1** opens at least up to 12 K (30 K) in the polycrystalline sample with ZFC maxima at 12 K, which is the highest ever known for any SIMs. Previous highest energy barrier and highest blocking temperature have been observed for heteroleptic {Tb(Pc)(Pc′)}^–^ (938 K)1*d* and {Tb_2_–N_2_^3–^} complexes (14 K),1*c* respectively, but both these complexes exhibit one of these two properties at the expense of the other. For the first time, an undiluted air-stable SIM has been shown to exhibit a hysteresis loops up to 12 K (30 K), while not compromising on the energy barrier. Thus, the combination of a large barrier height, blocking temperature and coercivity makes **1** as one of the best observed SIMs/SMMs to be reported to date (for a comparison, *cf.*Table 1).

This analysis highlights the importance of higher order symmetry and the co-ordination environment (strong axial ligands) in obtaining larger energy barriers. It appears that such pseudo-linear geometries around Ln^3+^ ions with other types of equatorial ligands may provide the key for unlocking even higher *U*_eff_ and *T*_B_. Search for such systems are currently underway.

## Experimental section

### Instruments and methods

Infrared spectra were recorded on a Perkin Elmer Spectrum One spectrometer using KBr diluted pellets. Elemental analyses were performed on Thermoquest Flash EA 1112 series CHNS Elemental analyzer. NMR spectra were recorded using a Bruker Advance DPX-400 spectrometer. The magnetic properties of **1**, **2** and **1′** were measured using a Quantum Design MPMS-XL SQUID magnetometer equipped with a 7 T magnet in the temperature range 2–300 K using polycrystalline powder samples. Magnetization measurements at a continuous sweep rate of 0.002 T s^–1^ were performed on a Quantum Design PPMS equipped with a VSM setup.

### Materials

Commercial grade solvents were purified by employing conventional procedures.^30^ The phosphonic diamide ligand ^*t*^BuPO(NH^i^Pr)_2_ was synthesized using a previously reported procedure.^11^ LnI_3_·*x*H_2_O were prepared from Ln_2_O_3_ (Alfa Aesar).

### General procedure for the synthesis of [L_2_Ln(H_2_O)_5_][I]_3_·L_2_(H_2_O)

To a solution of LnI_3_·*x*H_2_O (0.20 mmol) in methanol (20 mL) was added a solution of ^*t*^BuPO(NH^i^Pr)_2_ (0.264 mg, 1.2 mmol) in methanol (10 mL). The reaction mixture was stirred at 60 °C for 1 h before cooling down to room temperature. The solution was then filtered and kept for crystallization at ambient aerobic conditions. Pale yellow crystals were obtained by slow evaporation of the solvent. The product obtained was then thoroughly washed with toluene.

### [L_2_Dy(H_2_O)_5_][I]_3_·L_2_·(H_2_O)] (**1**)

Yield: 0.170 g (55%, based on ligand). Anal. calc. for C_40_H_112_DyI_3_N_8_O_10_P_4_: C, 31.35; H, 7.37; N, 7.31. Found: C, 31.65; H, 7.44; N, 7.04%. FT-IR (KBr, cm^–1^): 3379 (s), 3288 (br), 2969 (s), 2868 (m), 1624 (br), 1470 (m), 1420 (s), 1396 (m), 1385 (m), 1369 (m), 1311 (w), 1144 (vs), 1130 (vs), 1114 (vs), 1100 (vs), 1049 (s), 1025 (s), 907 (m), 886 (w), 829 (m), 722 (m), 655 (m), 545 (w).

### [L_2_Er(H_2_O)_5_][I]_3_·L_2_·(H_2_O)] (**2**)

Yield: 0.150 g (48%, based on ligand). Anal. calc. for C_40_H_112_ErI_3_N_8_O_10_P_4_: C, 31.25; H, 7.34; N, 7.29. Found: C, 31.46; H, 7.69; N, 7.16%. FT-IR (KBr, cm^–1^): 3384 (s), 3270 (br), 2968 (s), 2870 (m), 1619 (br), 1476 (m), 1464 (m), 1420 (s), 1386 (m), 1366 (m), 1312 (w), 1172 (vs), 1141 (vs), 1131 (vs), 1115 (vs), 1049 (s), 10 125 (s), 906 (w), 884 (m), 830 (m), 731 (w), 654 (w), 545 (w).

### [L_2_Y(H_2_O)_5_][I]_3_·L_2_·(H_2_O)] (**3**)

Yield: 0.160 g (54%, based on ligand). Anal. calc. for C_40_H_112_I_3_N_8_O_10_P_4_Y: C, 32.93; H, 7.74; N, 7.68. Found: C, 32.95; H, 8.00; N, 7.45%. FT-IR (KBr, cm^–1^): 3379 (s), 3292 (br), 2969 (s), 2873 (m), 1621 (br), 1470 (m), 1420 (s), 1388 (m), 1311 (w), 1144 (vs), 1130 (vs), 1113 (vs), 1107 (vs), 1049 (s), 1024 (s), 906 (w), 885 (w), 829 (w), 727 (w), 655 (w), 544 (w). ^1^H NMR (CD_3_CN, 400 MHz): *δ* 3.42 (br, 8H, NH), 3.22 (br, 10H, OH_2_), 3.14 (br, 8H, –C*H*(CH_3_)_2_), 1.20 (d, 24H, –NCH(C*H*_3_)_2_, *J* = 6.4 Hz), 1.20 (d, 24H, –NCH(C*H*_3_)_2_, *J* = 6.4 Hz), 1.14 (s, 18H, –C(C*H*_3_)_3_), 1.11 (s, 18H, –C(C*H*_3_)_3_). ^13^C NMR (CD_3_CN, 100 MHz): *δ* 44.28, 33.68, 32.74, 27.01, 26.99, 26.64, 26.60, 25.73. ^31^P NMR (CD_3_CN, 162 MHz): *δ* – 38.8 ppm.

### Preparation of [L_2_Dy_0.25_Y_0.75_(H_2_O)_5_][I]_3_·L_2_·(H_2_O)] (**1′**)

Analytically pure crystalline samples of **1** and **3** were accurately weighed in 1 : 3 molar ratio and dissolved in a minimum amount of dichloromethane and benzene. The clear solution obtained was vigorously stirred for 1 h and the solvent was slowly allowed to evaporate to obtain a crystalline powder. Anal. calc. for C_40_H_112_Dy_0.25_I_3_N_8_O_10_P_4_Y_0.75_: C, 32.52; H, 7.64; N, 7.59. Found: C, 32.46; H, 7.54; N, 7.61. FT-IR (KBr, cm^–1^): 3379 (s), 3291 (br), 2969 (s), 2868 (m), 1622 (br), 1476 (m), 1470 (m), 1421 (s), 1396 (m), 1381 (m), 1319 (w), 1144 (vs), 1130 (vs), 1115 (vs), 1105 (vs), 1049 (s), 1025 (s), 906 (w), 885 (w), 830 (w), 726 (w), 655 (w), 548 (w).

### Single-crystal X-ray crystallography

Suitable single crystals of **1–3** were selected and mounted on a Rigaku Saturn 724+ CCD diffractometer using paratone oil for unit cell determination and three-dimensional intensity data collection. Data integration and indexing was carried out using CrystalClear and CrystalStructure.^31^ The structures were solved using direct methods (SIR-97).^32^ Structure refinement and geometrical calculations were carried out using programs in the WinGX module.^33^ The final structure refinement was carried out using full least square methods on *F*^2^ using SHELXL-2014.^34^ Details of crystal data and structure refinement of the isomorphous compounds **1–3** are given in Table S1.†

### Magnetic studies

The samples were immobilised in an eicosane matrix to prevent torquing during the experiments. The data were corrected for the background diamagnetic contribution and the diamagnetic contributions of the compounds were corrected using Pascal's constants. Alternating current (ac) susceptibility measurements were performed with an oscillating ac field of 3.5 Oe oscillating at indicated frequencies between 0.1 and 1464 Hz.

### Computational details

*Ab initio* calculations have been carried out on the complexes **1** and **2** to compute the *g*-tensors and the energies of the Kramers doublet. All the calculations have been performed using MOLCAS 7.8^26^,^35^ quantum chemistry package. In this multi-configurational approach, relativistic approach has been treated based on Douglas–Kroll Hamiltonian. We have employed atomic natural (ANO-RCC) basis set for the calculations of *g*-tensors. The following contraction scheme have been employed: [8s7p5d3f2g1h] for Dy and Er, [3s2p] for N, [4s3p2d1f] for O, [6s5p2d] for I, [4s3p] for P, [3s2p] for C and [2s] for H. The ground-state atomic multiplicity of Dy^III^ is ^6^H_15/2_ which results in eight low-lying Kramers doublets. The CASSCF calculation comprises an active space of nine active electrons in the seven active orbitals (CAS (9,7)). With this active space, we have computed 21 sextets, 224 quartets and 264 doublet states. In the next step we have mixed these CASSCF computed spin-free states (21 sextets, 128 quartets and 98 doublets were considered due to hardware limitations) *via* the RASSI module to obtain the spin–orbit states. The ground state f-electron configuration for Er^III^ is 4f^11^ and this yields ^4^I_15/2_ multiplet as the ground state. First, we performed CASSCF calculations with an active space of eleven active electrons in seven 4f orbitals (11,7). With this active space, we have computed 35 quartets as well 112 doublet states in the CI (Configuration Interaction) procedure. After computing these excited states, we have mixed all these 35 quartets and 112 doublets using RASSI-SO module to compute the spin–orbit coupled states. In the last step we have used SINGLE_ANISO code^27^ implemented in the MOLCAS to compute the *g*-tensors of Dy^III^ and Er^III^ ions. Furthermore our computed molar magnetic susceptibility and molar magnetization have been computed and found to be nicely agreeing with experimental observations.

### DFT calculations

Density functional calculations have been performed using G09 code^36^ to analyse the NPA charges of complexes **1** and **2**. We have employed UB3LYP^37^ functional along with a CSDZ basis set^38^ on Dy, Er ions, SDD basis set^39^ on I and TZV basis set^40^ for the remainder of the atoms.

## Supplementary Material

Supplementary information

Crystal structure data

## References

[cit1] (g) Gatteschi D., Sessoli R. and Villain J., in Molecular Nanomagnets, Oxford University Press, 2006.

[cit2] Jiang S.-D., Wang B.-W., Sun H.-L., Wang Z.-M., Gao S. (2011). J. Am. Chem. Soc..

[cit3] Ungur L., Le Roy J. J., Korobkov I., Murugesu M., Chibotaru L. F. (2014). Angew. Chem., Int. Ed..

[cit4] Vincent R., Klyatskaya S., Ruben M., Wernsdorfer W., Balestro F. (2012). Nature.

[cit5] Ishikawa N., Sugita M., Ishikawa T., Koshihara S.-y., Kaizu Y. (2003). J. Am. Chem. Soc..

[cit6] Blagg R. J., Muryn C. A., McInnes E. J. L., Tuna F., Winpenny R. E. P. (2011). Angew. Chem., Int. Ed..

[cit7] Rajeshkumar T., Rajaraman G. (2012). Chem. Commun..

[cit8] Ungur L., Chibotaru L. F. (2011). Phys. Chem. Chem. Phys..

[cit9] Liu J.-L., Wu J.-Y., Huang G.-Z., Chen Y.-C., Jia J.-H., Ungur L., Chibotaru L. F., Chen X.-M., Tong M.-L. (2015). Sci. Rep..

[cit10] Zadrozny J. M., Xiao D. J., Atanasov M., Long G. J., Grandjean F., Neese F., Long J. R. (2013). Nat. Chem..

[cit11] Murugavel R., Pothiraja R. (2003). New J. Chem..

[cit12] Llunell M., Casanova D., Cirera J., Bofill J., Alemany P. and Alvarez S., SHAPE (Version 2.1), Barcelona, 2013.

[cit13] Le Roy J. J., Ungur L., Korobkov I., Chibotaru L. F., Murugesu M. (2014). J. Am. Chem. Soc..

[cit14] Pedersen K. S., Ungur L., Sigrist M., Sundt A., Schau-Magnussen M., Vieru V., Mutka H., Rols S., Weihe H., Waldmann O., Chibotaru L. F., Bendix J., Dreiser J. (2014). Chem. Sci..

[cit15] Meihaus K. R., Long J. R. (2013). J. Am. Chem. Soc..

[cit16] Branzoli F., Carretta P., Filibian M., Zoppellaro G., Graf M. J., Galan-Mascaros J. R., Fuhr O., Brink S., Ruben M. (2009). J. Am. Chem. Soc..

[cit17] Gonidec M., Amabilino D. B., Veciana J. (2012). Dalton Trans..

[cit18] Ishikawa N., Sugita M., Tanaka N., Ishikawa T., Koshihara S.-y., Kaizu Y. (2004). Inorg. Chem..

[cit19] Takamatsu S., Ishikawa T., Koshihara S.-y., Ishikawa N. (2007). Inorg. Chem..

[cit20] Gonidec M., Luis F., Vílchez À., Esquena J., Amabilino D. B., Veciana J. (2010). Angew. Chem., Int. Ed..

[cit21] Pugh T., Tuna F., Ungur L., Collison D., McInnes E. J. L., Chibotaru L. F., Layfield R. A. (2015). Nat Commun.

[cit22] Yi X., Bernot K., Pointillart F., Poneti G., Calvez G., Daiguebonne C., Guillou O., Sessoli R. (2012). Chem.–Eur. J..

[cit23] Tuna F., Smith C. A., Bodensteiner M., Ungur L., Chibotaru L. F., McInnes E. J. L., Winpenny R. E. P., Collison D., Layfield R. A. (2012). Angew. Chem., Int. Ed..

[cit24] Meihaus K. R., Rinehart J. D., Long J. R. (2011). Inorg. Chem..

[cit25] (f) Ungur L. and Chibotaru L. F., in Lanthanides and Actinides in Molecular Magnetism, ed. R. Layfield and M. Murugesu, Wiley, New Jersey, 2015, ch. 6, pp. 153–184.

[cit26] Aquilante F., De Vico L., Ferre N., Ghigo G., Malmqvist P. A., Neogrady P., Pedersen T. B., Pitonak M., Reiher M., Roos B. O., Serrano-Andres L., Urban M., Veryazov V., Lindh R. (2010). J. Comput. Chem..

[cit27] Chibotaru L. and Ungur L., The computer programs SINGLE_ANISO and POLY_ANISO, University of Leuven, 2006.

[cit28] Lines M. E. (1971). J. Chem. Phys..

[cit29] Chen Y.-C., Liu J.-L., Ungur L., Liu J., Li Q.-W., Wang L.-F., Ni Z.-P., Chibotaru L. F., Chen X.-M., Tong M.-L. (2016). J. Am. Chem. Soc..

[cit30] Armarego W. L. F. and Perrin D. D., Purification of laboratory chemicals, Butterworth Heinemann, Oxford, Boston, 1996.

[cit31] CrystalClear, Version-SM Expert 2.0 r4, 2009 and CrystalStructure, Version 4.0, Rigaku, 2010, Rigaku Americas and Rigaku, The Woodlands, Texas, USA and Rigaku Corporation, Tokyo, Japan.

[cit32] Altomare A., Burla M. C., Camalli M., Cascarano G. L., Giacovazzo C., Guagliardi A., Moliterni A. G. G., Polidori G., Spagna R. (1999). J. Appl. Crystallogr..

[cit33] Farrugia L. (2012). J. Appl. Crystallogr..

[cit34] Sheldrick G. (2015). Acta Crystallogr., Sect. C: Struct. Chem..

[cit35] Veryazov V., Widmark P.-O., Serrano-Andrés L., Lindh R., Roos B. O. (2004). Int. J. Quantum Chem..

[cit36] Frisch M. J., Trucks G. W., Schlegel H. B., Scuseria G. E., Robb M. A., Cheeseman J. R., Scalmani G., Barone V., Mennucci B., Petersson G. A., Nakatsuji H., Caricato M., Li X., Hratchian H. P., Izmaylov A. F., Bloino J., Zheng G., Sonnenberg J. L., Hada M., Ehara M., Toyota K., Fukuda R., Hasegawa J., Ishida M., Nakajima T., Honda Y., Kitao O., Nakai H., Vreven T., Montgomery, Jr. J. A., Peralta J. E., Ogliaro F., Bearpark M., Heyd J. J., Brothers E., Kudin K. N., Staroverov V. N., Kobayashi R., Normand J., Raghavachari K., Rendell A., Burant J. C., Iyengar S. S., Tomasi J., Cossi M., Rega N., Millam J. M., Klene M., Knox J. E., Cross J. B., Bakken V., Adamo C., Jaramillo J., Gomperts R., Stratmann R. E., Yazyev O., Austin A. J., Cammi R., Pomelli C., Ochterski J., Martin R. L., Morokuma K., Zakrzewski V. G., Voth G. A., Salvador P., Dannenberg J. J., Dapprich S., Daniels A. D., Farkas O., Foresman J. B., Ortiz J. V., Cioslowski J. and Fox D. J., GAUSSIAN 09, Gaussian, Inc., Wallingford, CT, 2009.

[cit37] Stephens P. J., Devlin F. J., Chabalowski C. F., Frisch M. J. (1994). J. Phys. Chem..

[cit38] Cundari T. R., Stevens W. J. (1993). J. Chem. Phys..

[cit39] Bergner A., Dolg M., Küchle W., Stoll H., Preuß H. (1993). Mol. Phys..

[cit40] Schäfer A., Huber C., Ahlrichs R. (1994). J. Chem. Phys..

