# Supplementary material for: An air-stable Dy(iii) single-ion magnet with high anisotropy barrier and blocking temperature
Source: Chem Sci. 2016 Apr 13;7(8):5181–91. doi: 10.1039/c6sc00279j (PMC6020529; doi:10.1039/c6sc00279j)
Supplement: Supplementary file 1 [file SC-007-C6SC00279J-s001.pdf]

Electronic Supplementary Material (ESI) for Chemical Science.
This journal is © The Royal Society of Chemistry 2016

An Air-Stable Dy(III) Single-Ion Magnet with High Anisotropy Barrier and Blocking Temperature

Sandeep K. Gupta, Thayalan Rajeshkumar, Gopalan Rajaraman,* Ramaswamy Murugavel*

Department of Chemistry, Indian Institute of Technology Bombay, Mumbai-400 076, India

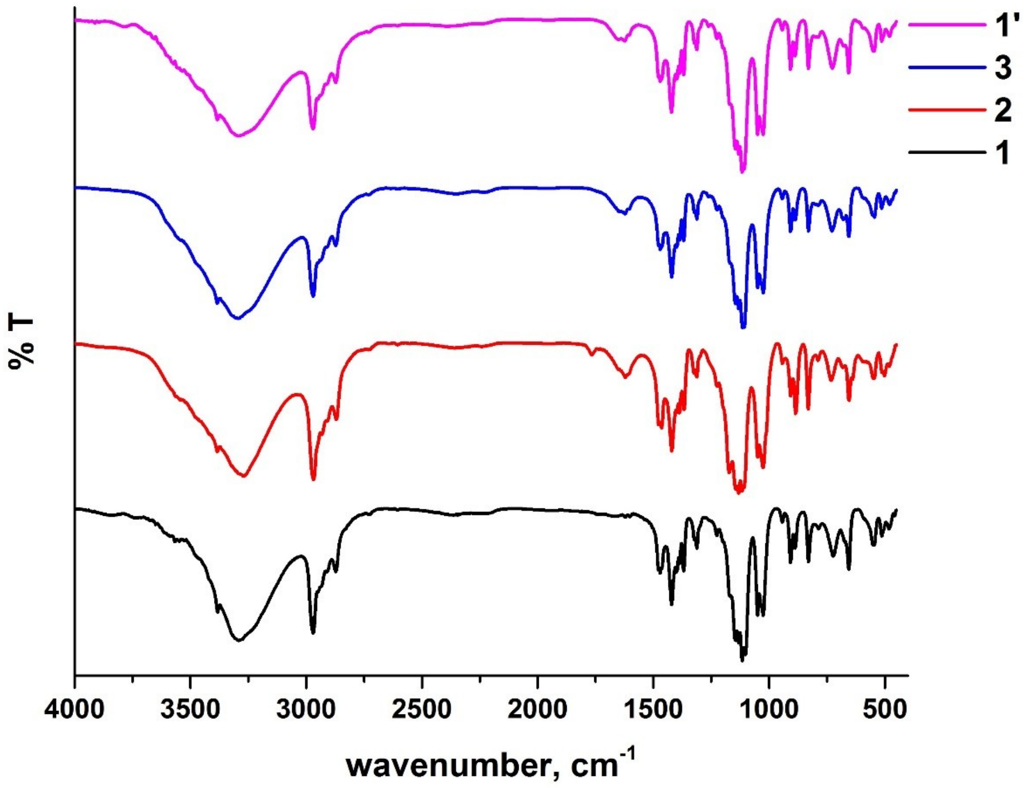

Figure S1. FTIR spectra of the complexes as disc diluted in KBr.

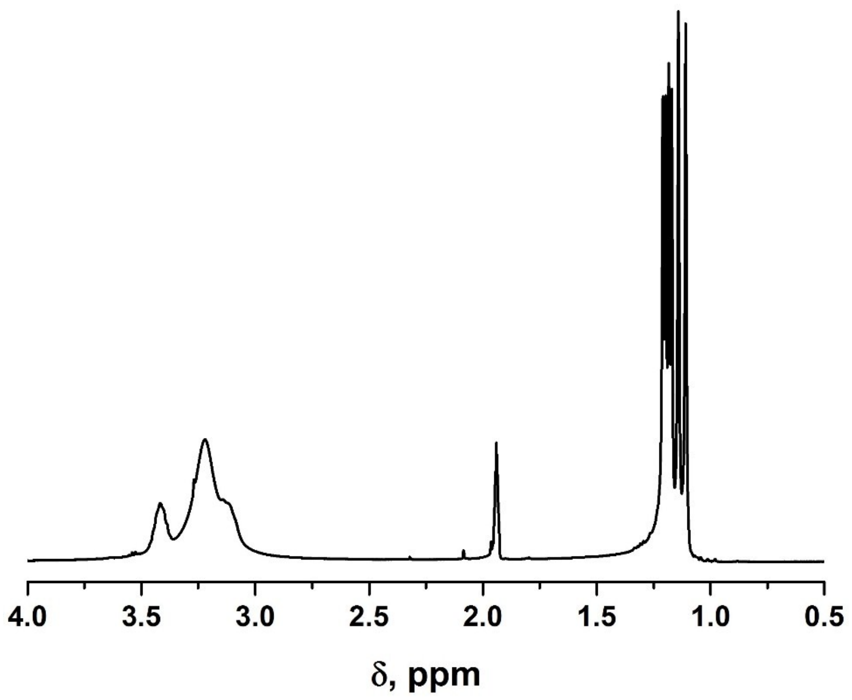

Figure S2. ^{1}H NMR spectrum of **3** (CD_3CN).

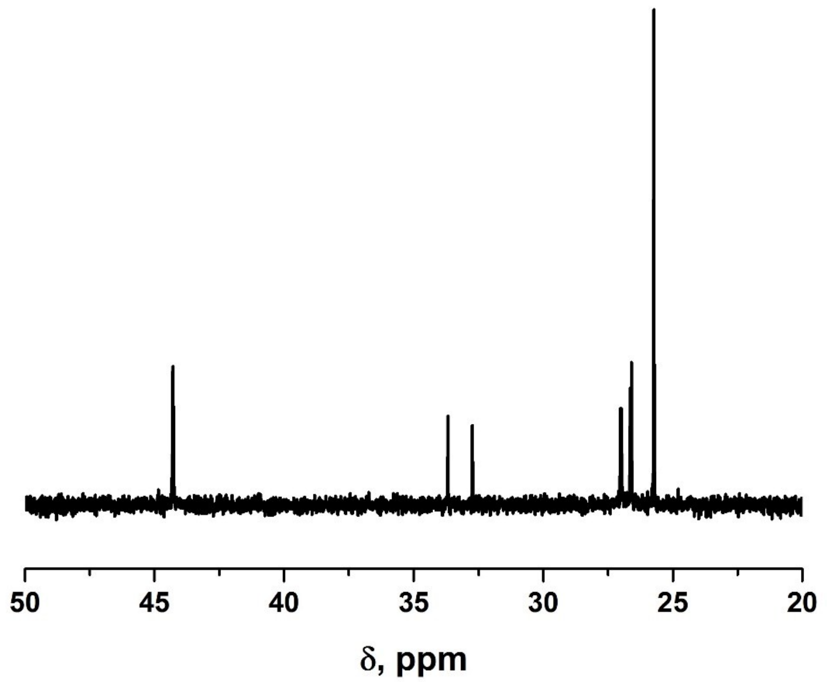

Figure S3. ^{13}C NMR spectrum of **3** (CD_3CN).

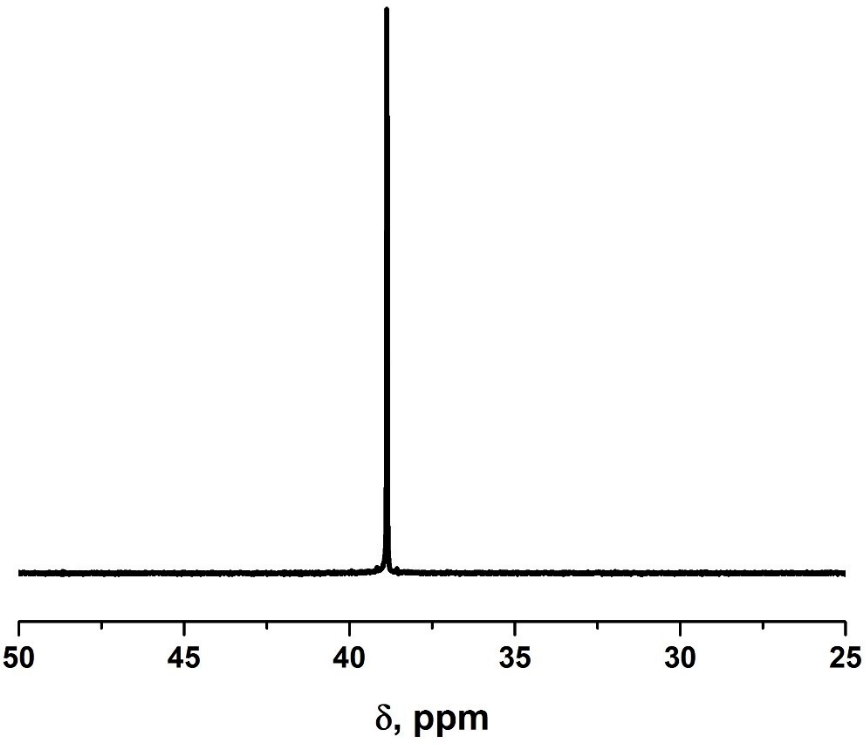

Figure S4. ^{31}P NMR spectrum of **3** (CD_3CN).

Table S1. Crystallographic Refinement Data for **1-3**.

**Compound**	**1**	**2**	**3**
Empirical formula	$C_{40}H_{112}DyI_3N_8O_{10}P_4$	$C_{40}H_{112}ErI_3N_8O_{10}P_4$	$C_{40}H_{112}I_3N_8O_{10}P_4Y$
Formula weight	1532.45	1537.21	1458.86
Temperature (K)	120(2)	120(2)	120(2)
Wavelength (Å)	0.71075	0.71075	0.71075
Crystal system	Triclinic	Triclinic	Triclinic
Space group	P -1	P -1	P -1
a (Å)	13.7628(10)	13.7370(8)	13.7506(13)
b (Å)	14.6969(13)	14.6858(14)	14.727(2)
c (Å)	20.612(3)	20.6030(17)	20.641(2)
α (°)	92.199(10)	92.107(9)	92.293(14)
β (°)	108.292(8)	108.177(7)	108.245(10)
γ (°)	115.120(8)	115.121(12)	114.902(8)
Volume ($Å^3$)	3511.9(7)	3505.6(6)	3528.3(7)
Z	2	2	2
Density calcd. (mg/m^3)	1.449	1.456	1.373
μ (mm^{-1})	2.520	2.655	2.275
F(000)	1546	1550	1492
Crystal size (mm^3)	0.16 x 0.12 x 0.06	0.24 x 0.19 x 0.17	0.18 x 0.14 x 0.06
θ range (°)	2.473 to 25.250	2.375 to 25.249	2.205 to 25.250
Reflections collected	27150	26936	27431
Independent reflections	12555 [R(int) = 0.0245]	12526 [R(int) = 0.0242]	12633 [R(int) = 0.0233]
Data / restraints / parameters	12555 / 9 / 684	12526 / 10 / 703	12633 / 2 / 636
Goodness-of-fit on F2	1.09	1.079	1.06
Final R indices [I>2sigma(I)]	R1 = 0.0318, wR2 = 0.0658	R1 = 0.0256, wR2 = 0.0622	R1 = 0.0336, wR2 = 0.0762
R indices (all data)	R1 = 0.0335, wR2 = 0.0667	R1 = 0.0262, wR2 = 0.0625	R1 = 0.0360, wR2 = 0.0775
Largest diff. peak and hole (e.$Å^{-3}$)	1.336 and -1.403	1.426 and -1.342	1.294 and -1.516

Table S2. Selected bond distances (Å) and angles (°) in compound **1**.

Dy(1)-O(1)	2.208(2)	O(2)-Dy(1)-O(1)	175.14(9)	O(2)-Dy(1)-O(8)	89.46(10)
Dy(1)-O(2)	2.203(2)	P(1)-O(1)-Dy(1)	149.72(15)	O(2)-Dy(1)-O(9)	85.96(10)
Dy(1)-O(5)	2.366(2)	P(2)-O(2)-Dy(1)	155.17(16)	O(5)-Dy(1)-O(8)	143.70(9)
Dy(1)-O(6)	2.363(3)	O(1)-Dy(1)-O(5)	87.71(9)	O(6)-Dy(1)-O(5)	71.67(9)
Dy(1)-O(7)	2.355(3)	O(1)-Dy(1)-O(6)	91.89(9)	O(6)-Dy(1)-O(8)	144.50(10)
Dy(1)-O(8)	2.375(3)	O(1)-Dy(1)-O(7)	95.42(10)	O(7)-Dy(1)-O(5)	144.51(9)
Dy(1)-O(9)	2.356(3)	O(1)-Dy(1)-O(8)	87.82(10)	O(7)-Dy(1)-O(6)	72.90(10)
O(1)-P(1)	1.511(3)	O(1)-Dy(1)-O(9)	89.40(10)	O(7)-Dy(1)-O(8)	71.79(10)
O(2)-P(2)	1.509(3)	O(2)-Dy(1)-O(5)	92.11(9)	O(7)-Dy(1)-O(9)	144.73(10)
O(3)-P(3)	1.507(3)	O(2)-Dy(1)-O(6)	92.67(10)	O(9)-Dy(1)-O(5)	70.43(9)
O(4)-P(4)	1.500(3)	O(2)-Dy(1)-O(7)	87.55(10)	O(9)-Dy(1)-O(6)	141.98(10)
				O(9)-Dy(1)-O(8)	73.52(10)

Table S3. Selected bond distances (Å) and angles (°) in compound **2**.

Er(1)-O(1)	2.1984(19)	O(2)-Er(1)-O(1)	174.88(7)	O(2)-Er(1)-O(9)	85.87(8)
Er(1)-O(2)	2.1910(19)	P(1)-O(1)-Er(1)	149.96(13)	O(5)-Er(1)-O(6)	71.68(8)
Er(1)-O(5)	2.334(2)	P(2)-O(2)-Er(1)	155.06(13)	O(5)-Er(1)-O(8)	143.77(8)
Er(1)-O(6)	2.335(2)	O(1)-Er(1)-O(5)	88.21(8)	O(6)-Er(1)-O(8)	144.44(8)
Er(1)-O(7)	2.325(2)	O(1)-Er(1)-O(6)	92.03(8)	O(7)-Er(1)-O(5)	144.33(8)
Er(1)-O(8)	2.343(2)	O(1)-Er(1)-O(7)	94.63(8)	O(7)-Er(1)-O(6)	72.69(8)
Er(1)-O(9)	2.331(2)	O(1)-Er(1)-O(8)	87.71(8)	O(7)-Er(1)-O(8)	71.89(8)
O(1)-P(1)	1.508(2)	O(1)-Er(1)-O(9)	89.29(8)	O(7)-Er(1)-O(9)	144.80(9)
O(2)-P(2)	1.508(2)	O(2)-Er(1)-O(5)	91.76(8)	O(9)-Er(1)-O(5)	70.63(9)
O(3)-P(3)	1.503(2)	O(2)-Er(1)-O(6)	92.81(8)	O(9)-Er(1)-O(6)	142.23(9)
O(4)-P(4)	1.506(2)	O(2)-Er(1)-O(7)	88.38(8)	O(9)-Er(1)-O(8)	73.34(9)
		O(2)-Er(1)-O(8)	89.31(8)		

Table S4. Selected bond distances (Å) and angles (°) in compound **3**.

O(1)-Y(1)	2.206(2)	O(2)-Y(1)-O(1)	175.02(8)	O(2)-Y(1)-O(9)	86.03(8)
O(2)-Y(1)	2.201(2)	P(1)-O(1)-Y(1)	150.05(13)	O(5)-Y(1)-O(8)	143.74(8)
O(5)-Y(1)	2.3478(14)	P(2)-O(2)-Y(1)	155.71(14)	O(6)-Y(1)-O(5)	71.8
O(6)-Y(1)	2.3421(13)	O(1)-Y(1)-O(5)	88.15(8)	O(6)-Y(1)-O(7)	72.6
O(7)-Y(1)	2.3428(15)	O(1)-Y(1)-O(6)	92.12(8)	O(6)-Y(1)-O(8)	144.40(8)
O(8)-Y(1)	2.355(2)	O(1)-Y(1)-O(7)	94.51(8)	O(6)-Y(1)-O(9)	142.23(6)
O(9)-Y(1)	2.3465(18)	O(1)-Y(1)-O(8)	87.76(8)	O(7)-Y(1)-O(5)	144.38(6)
O(1)-P(1)	1.514(2)	O(1)-Y(1)-O(9)	89.31(8)	O(7)-Y(1)-O(8)	71.88(8)
O(2)-P(2)	1.507(2)	O(2)-Y(1)-O(5)	91.95(8)	O(7)-Y(1)-O(9)	144.84(7)
O(3)-P(3)	1.502(2)	O(2)-Y(1)-O(6)	92.64(8)	O(9)-Y(1)-O(5)	70.57(7)
O(4)-P(4)	1.503(2)	O(2)-Y(1)-O(7)	88.32(8)	O(9)-Y(1)-O(8)	73.37(9)
		O(2)-Y(1)-O(8)	89.23(9)		

Table S5. SHAPE measures of seven co-ordinate relative to structures of **1**.

Shape	Symmetry	Deviation
Johnson elongated triangular pyramid	C_{3v}	22.964
Johnson pentagonal bipyramid	D_{5h}	2.677
Capped trigonal prism	C_{2v}	5.795
Capped octahedron	C_{3v}	7.607
Pentagonal bipyramid	D_{5h}	0.224
Hexagonal pyramid	C_{6v}	24.648
Heptagon	D_{7h}	33.758

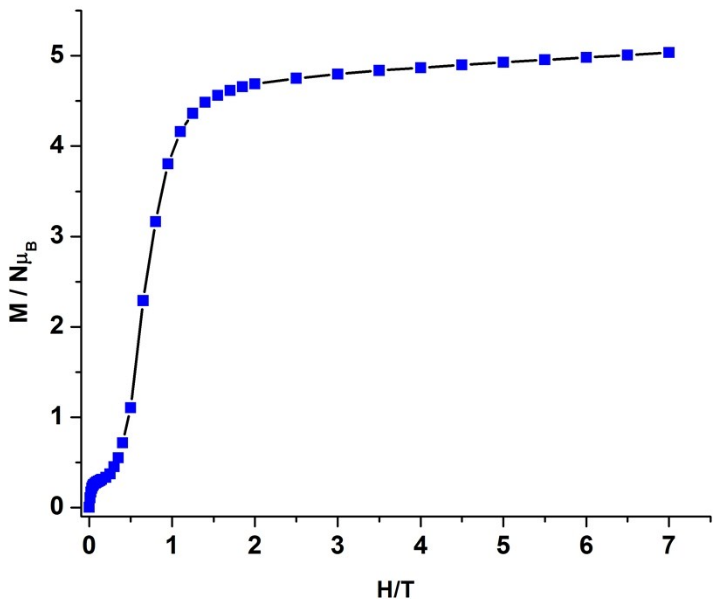

Figure S5. Field dependence of magnetization plot for **1** at 1.8 K.

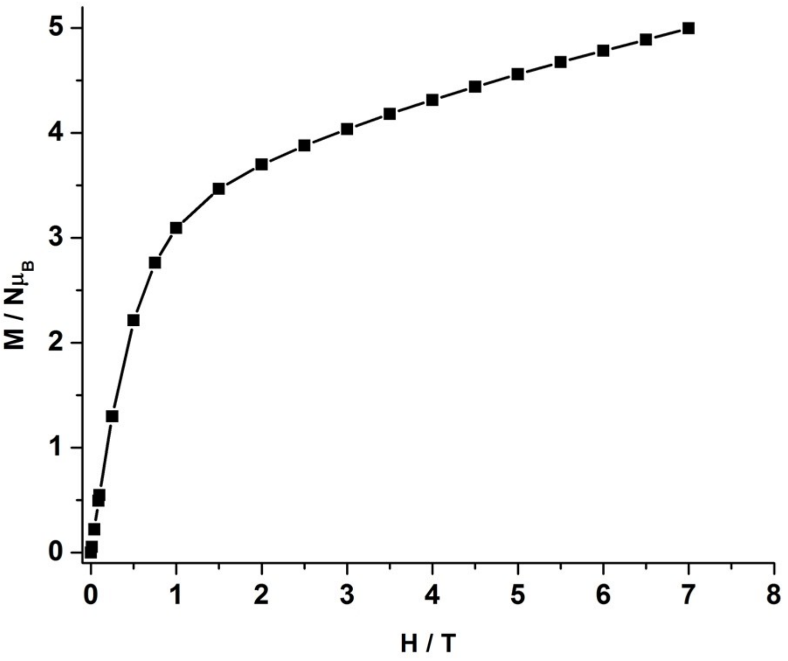

Figure S6. Field dependence of magnetization plot for **2** at 2.0 K.

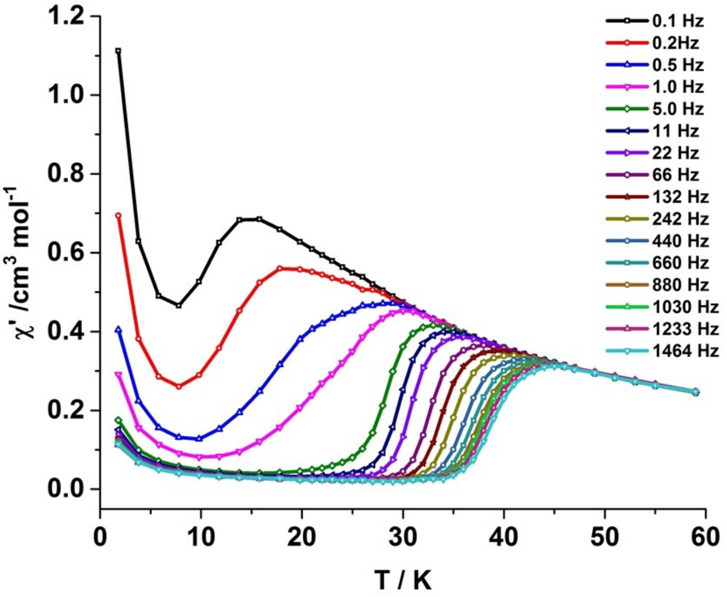

Figure S7. In-phase (χ_M') component of the ac susceptibility measured in an oscillating ac field of 3.5 Oe under zero applied dc field for complex **1**.

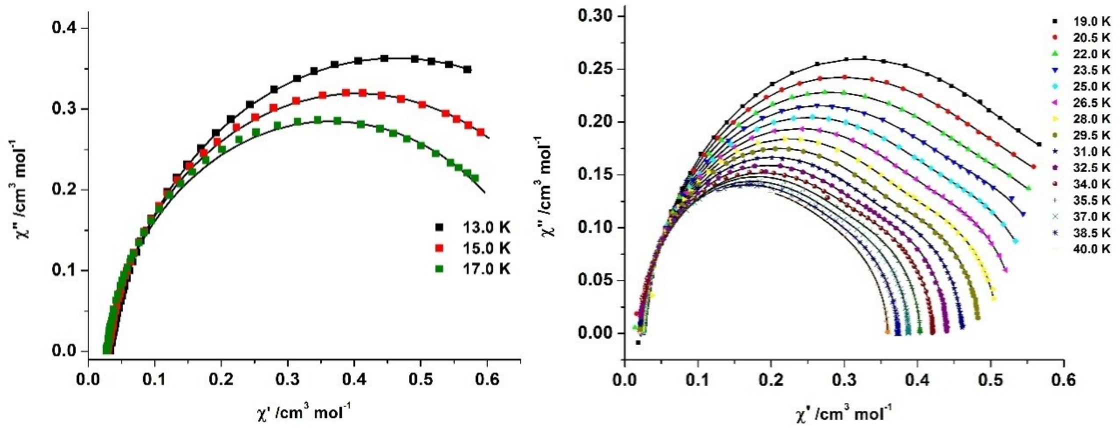

Figure S8. Plots of χ_M'' vs χ_M' at indicated temperatures. Solid lines are the fits with the Debye functions.

Fitting of the relaxation time with multiple process for 1

Both Arrhenius (ln τ versus T^{-1}) and Cole-Cole (χ_M'' vs χ_M') plots for **1** indicate the presence of multiple relaxation process. Hence the data were treated with the various relaxation processes reported in the literature with the following equation (1),

$$1/\tau = 1/\tau_{QTM} + AH^2T + CT^n + \tau_o^{-1} \exp\left(U_{eff}/k_BT\right) \quad ....(1)$$

where the first term corresponds to the relaxation process via quantum tunneling pathway, the second term models the direct process, the third term corresponds to the relaxation via Raman process, and the fourth term accounts for the Orbach relaxation pathway. Many fits were tried using a number of variable parameters in the equation. However, all the terms were not found to be necessary for fitting of the Arrhenius plot. Further the curvature in the plot (10 K- 30 K) cannot be attributed to QTM. In case of **1**, the linear dependence of ln τ with temperature deviates below 30 K as shown in Figure S9. The linear fit (indicated in dashed blue line) corresponds only to the Orbach relaxation pathway. The best fit for the Arrhenius plot could be obtained considering the Orbach and Raman relaxation process with the value of the Raman

exponent *n,* closer to 3. The values obtained from the best fit are n = 3 (T) and C = 0.000692 s^{-1} K^{-3} (T) (Figure S9) and n = 3 (ν) and C = 0.000430 s^{-1} K^{-3} (ν) (Figure S10).

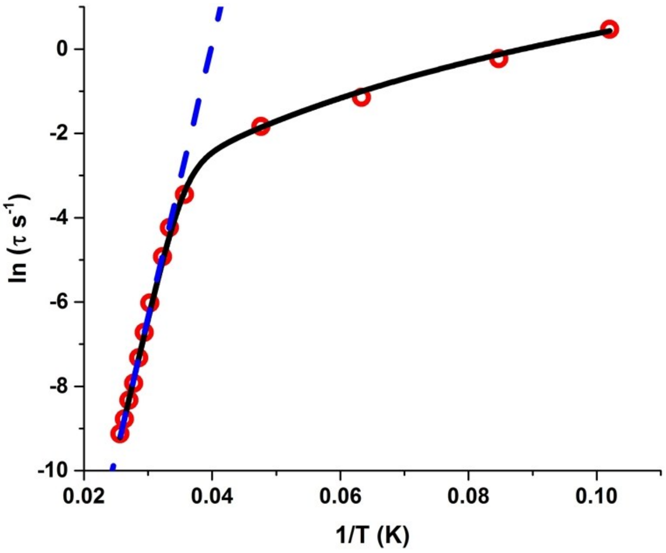

Figure S9. Plot of the relaxation time τ (T) (logarithmic scale) versus T^{-1} for **1**; the dashed blue line corresponds represents fitting to the Orbach relaxation process (U_{eff} = 651.0 K and a pre-exponential factor (τ_o) = 5.63 x 10^{-12} s) and the solid black line represents the best fitting to the multiple relaxation process.

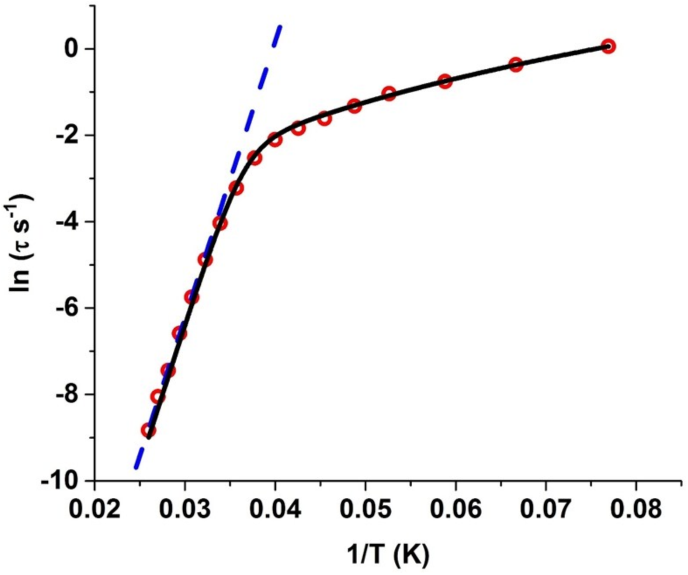

Figure S10. Plot of the relaxation time τ (ν) (logarithmic scale) versus T^{-1} for **1**; the dashed blue line corresponds represents fitting to the Orbach relaxation process (U_{eff} = 640.0 K and a pre-exponential factor (τ_o) = 9.16 x 10^{-12} s) and the solid black line represents the best fitting to the multiple relaxation process.

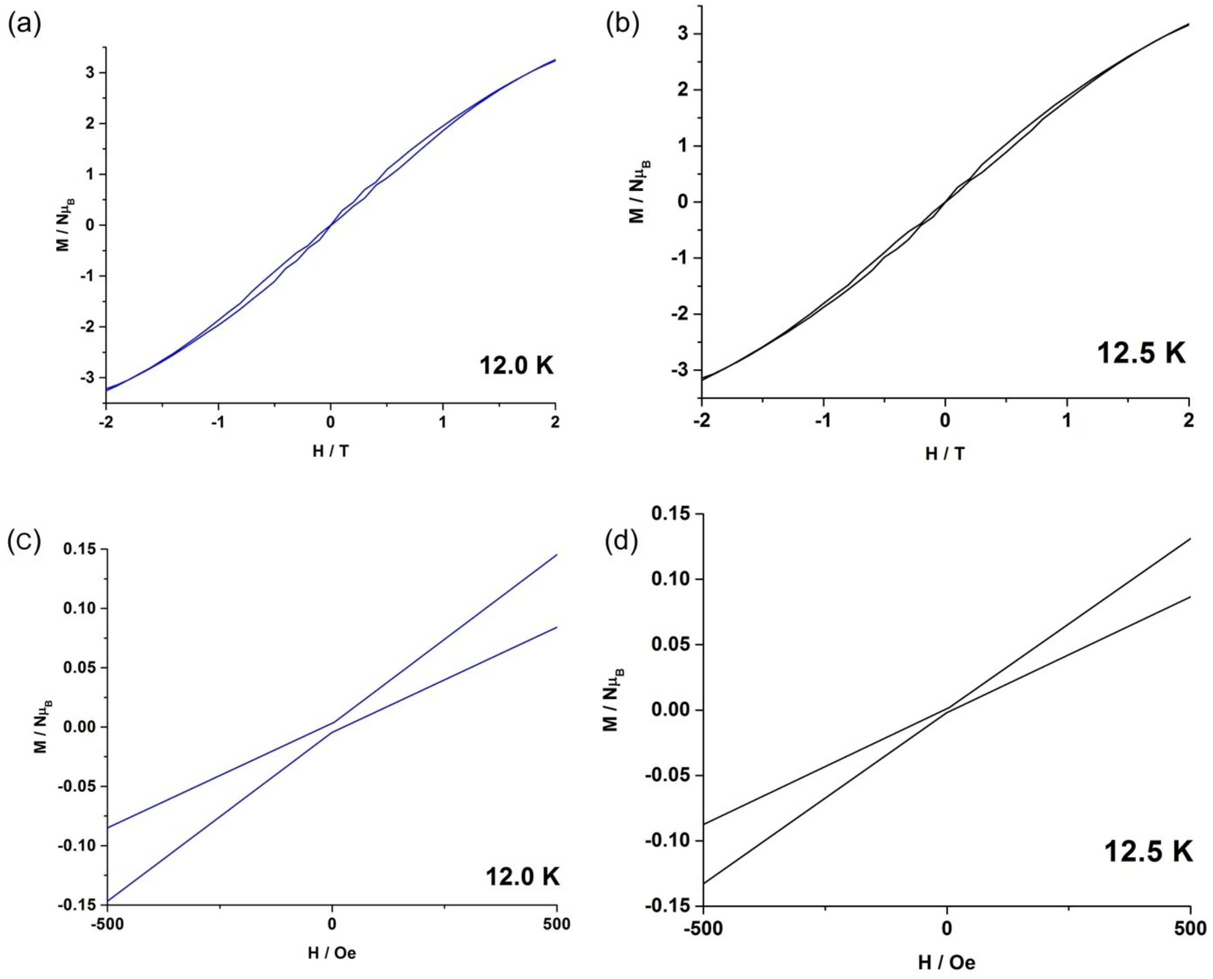

Figure S11 (a-d). Field dependence of magnetization plot for **1** at indicated temperatures at an average sweep rate of 0.0018 T s^{-1}.

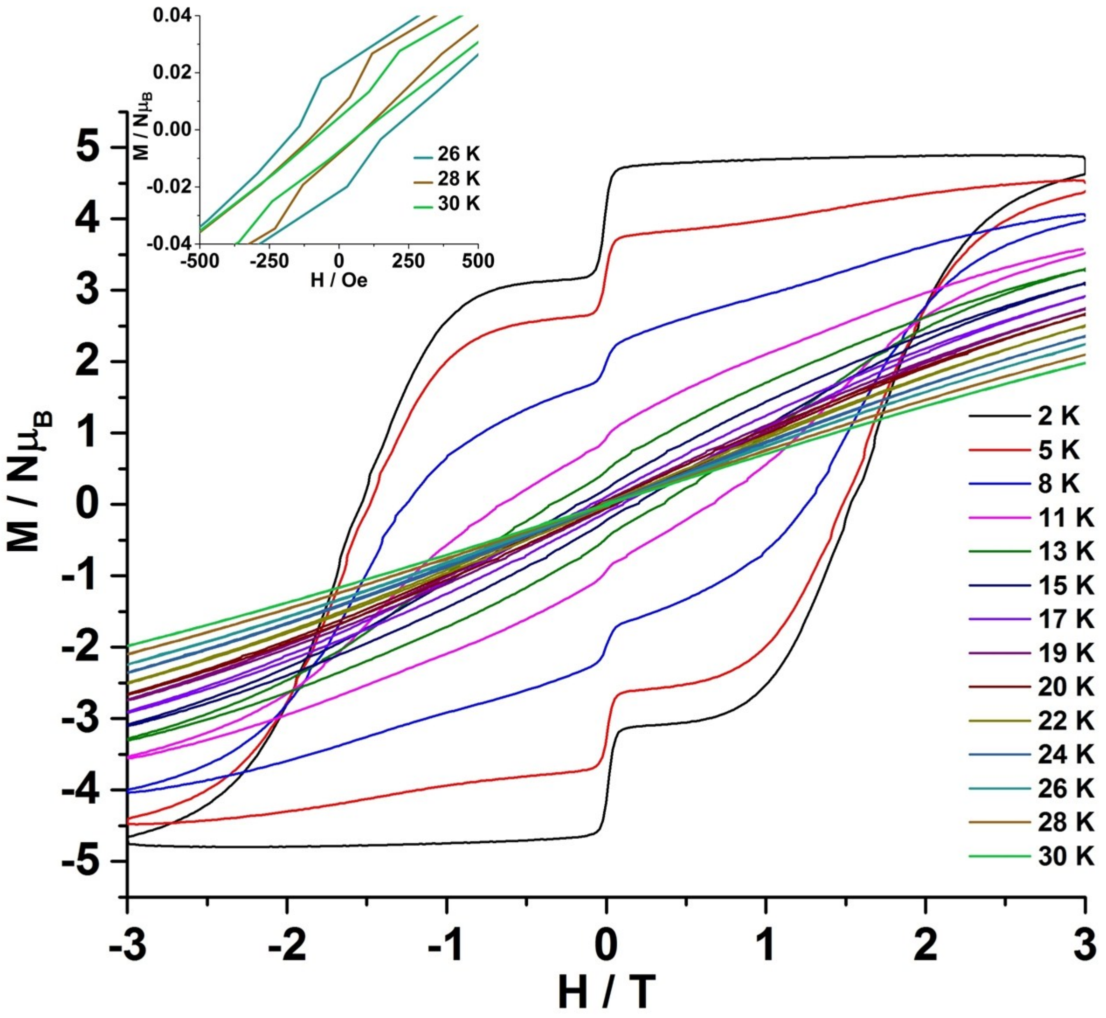

Figure S12. Field dependence of magnetization plot for **1** at indicated temperatures at an continuous sweep rate of 0.02 T s^{-1} .

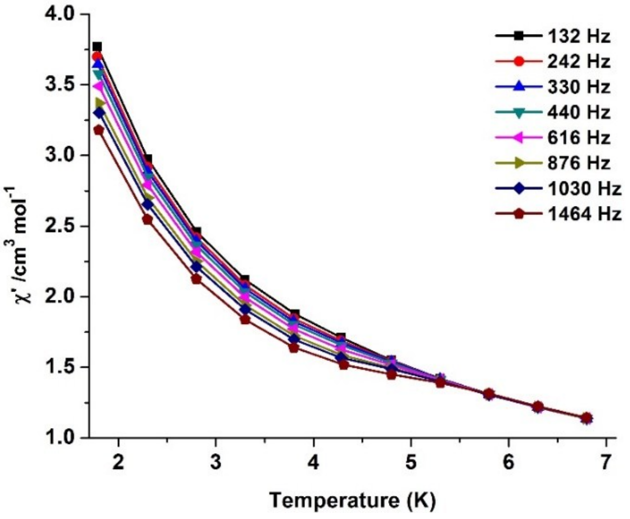

Figure S13. In-phase (χ_M') component of the ac susceptibility measured in an oscillating ac field of 3.5 Oe under zero applied dc field for complex **2.**

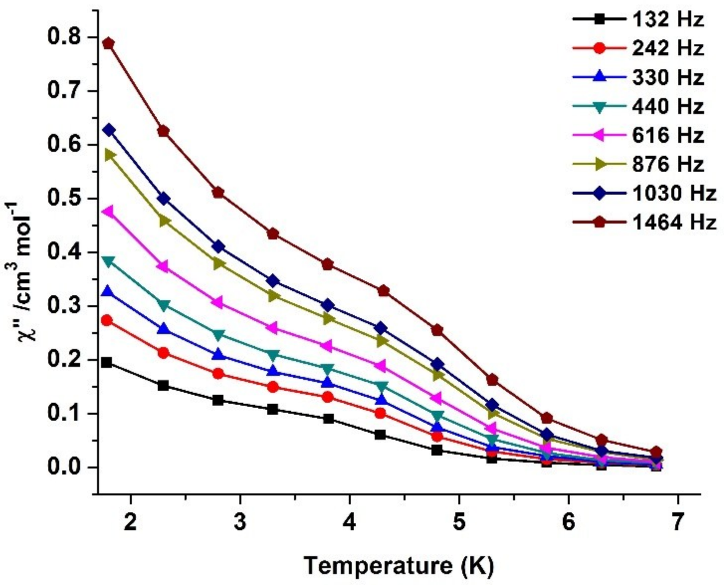

Figure S14. Out-of-phase (χ_M'') component of the ac susceptibility measured in an oscillating ac field of 3.5 Oe under zero applied dc field for complex **2**.

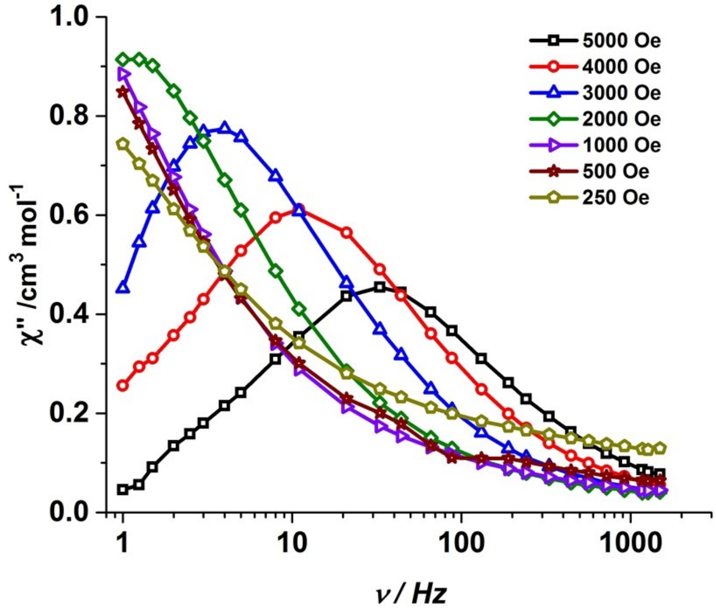

Figure S15. Frequency dependence of the out-of-phase (χ_M'') component of the ac susceptibility measured in an oscillating ac field of 3.5 Oe under various applied dc field for complex **2**.

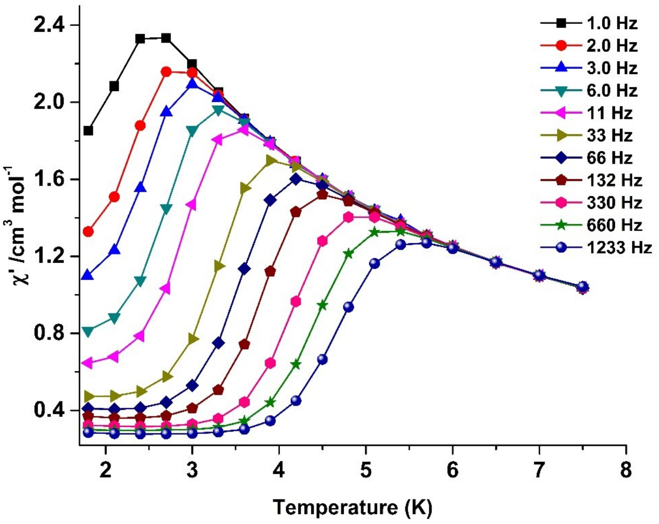

Figure S16. In-phase (χ_M') component of the ac susceptibility measured in an oscillating ac field of 3.5 Oe under an applied dc field of 2000 Oe for complex **2.**

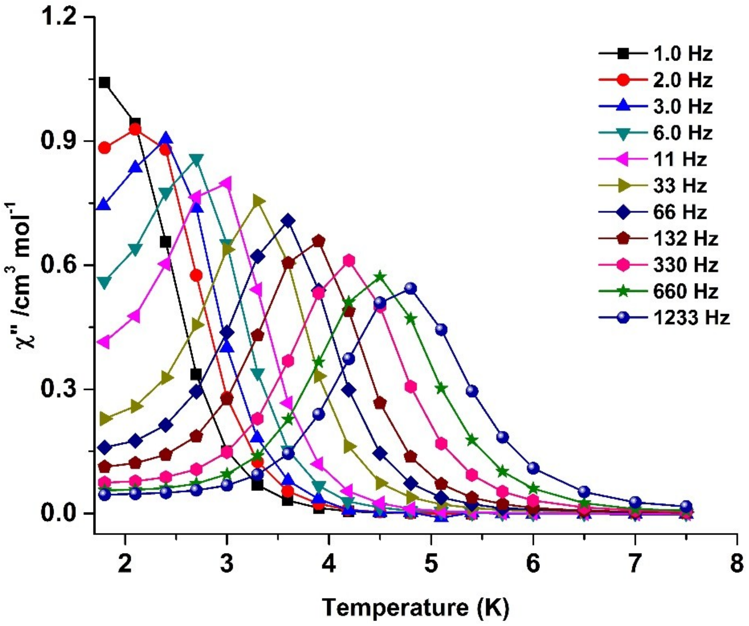

Figure S17. Out-of-phase (χ_M'') component of the ac susceptibility measured in an oscillating ac field of 3.5 Oe under an applied dc field of 2000 Oe for complex **2.**

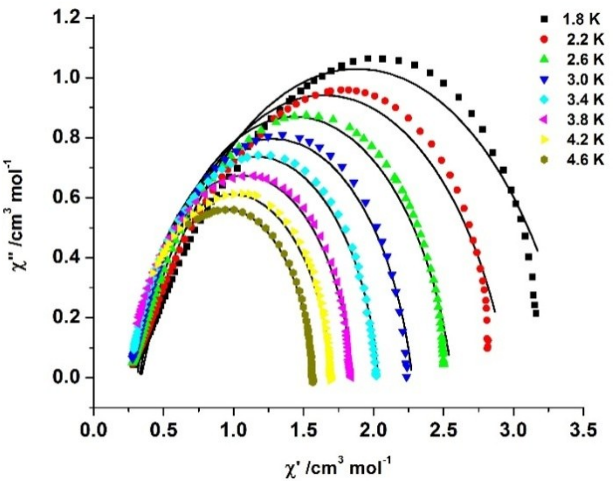

Figure S18. Plots of χ_M'' vs χ_M' at indicated temperatures for **2**. Solid lines are the fits with the Debye model considering only one relaxation process where it deviates considerably at lower temperatures indicating multiple relaxation process (0.06 < α < 0.25).

Fitting of the relaxation time with multiple process for 2

As in case of **1**, both Arrhenius (ln τ versus T^{-1}) and Cole-Cole (χ_M'' vs χ_M') plots for **2** indicate the presence of multiple relaxation process. Hence these data were treated with the various relaxation processes reported in the literature with the following equation (1),

$$1/\tau = 1/\tau_{QTM} + AH^2T + CT^n + \tau_o^{-1} \exp(U_{eff}/k_BT) \quad ....(1)$$

where the terms are explained above. Contrary to **1**, the best fit to the curvature in **2**, could be obtained utilizing three relaxation processes *viz.* the quantum tunneling, Raman and Orbach pathways, respectively. The values obtained from the best fits are: $1/\tau_{QTM} = 1.8715\ s^{-1}$ (T), n = 5 (T) and C = 0.2361 $s^{-1}\ K^{-5}$ (T) and $1/\tau_{QTM} = 2.8093\ s^{-1}$ (ν), n = 5 (ν) and C = 0.1456 $s^{-1}\ K^{-5}$ (ν).

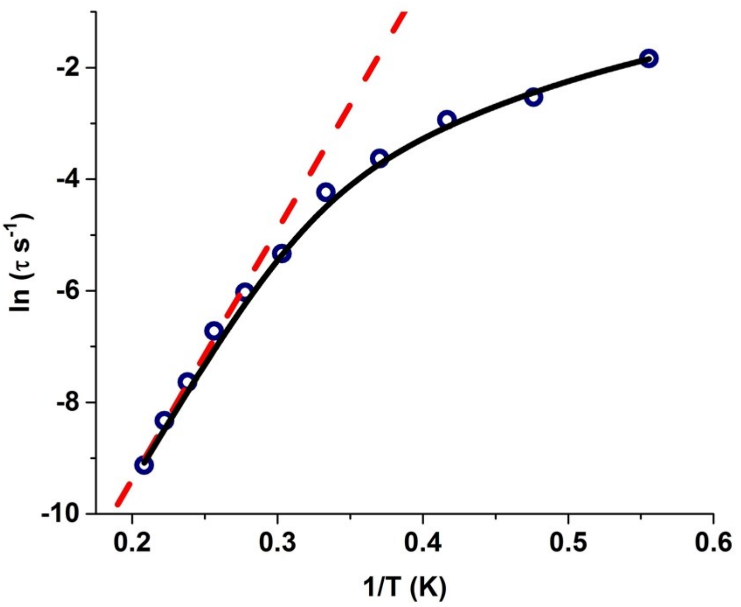

Figure S19. Plot of the relaxation time τ (T) (logarithmic scale) versus T^{-1} for **1**; the dashed red line corresponds represents fitting to the Orbach relaxation process and the solid black line represents the best fitting to the multiple relaxation process (U_{eff} = 44.7 K and a pre-exponential factor (τ_o) = 1.08 x 10^{-8} s).

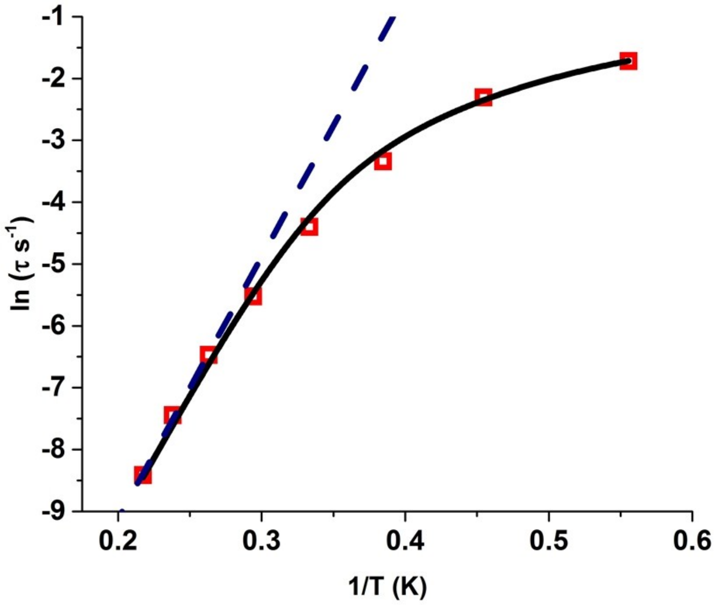

Figure S20. Plot of the relaxation time τ (ν) (logarithmic scale) versus T^{-1} for **2**; the dashed blue line corresponds represents fitting to the Orbach relaxation process and the solid black line represents the best fitting to the multiple relaxation process (U_{eff} = 42.4 K and a pre-exponential factor (τ_o) = 2.27 x 10^{-8} s).

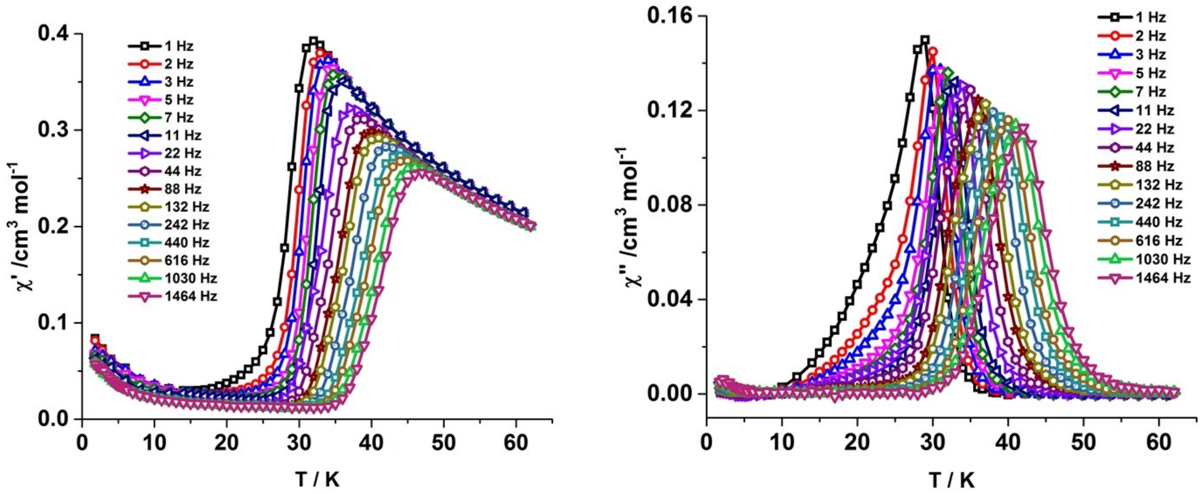

Figure S21. In-phase (χ_M') component (left) and out-of-phase (χ_M'') component (right) of the ac susceptibility measured in an oscillating ac field of 3.5 Oe under an applied dc field of 2000 Oe for complex **1.**

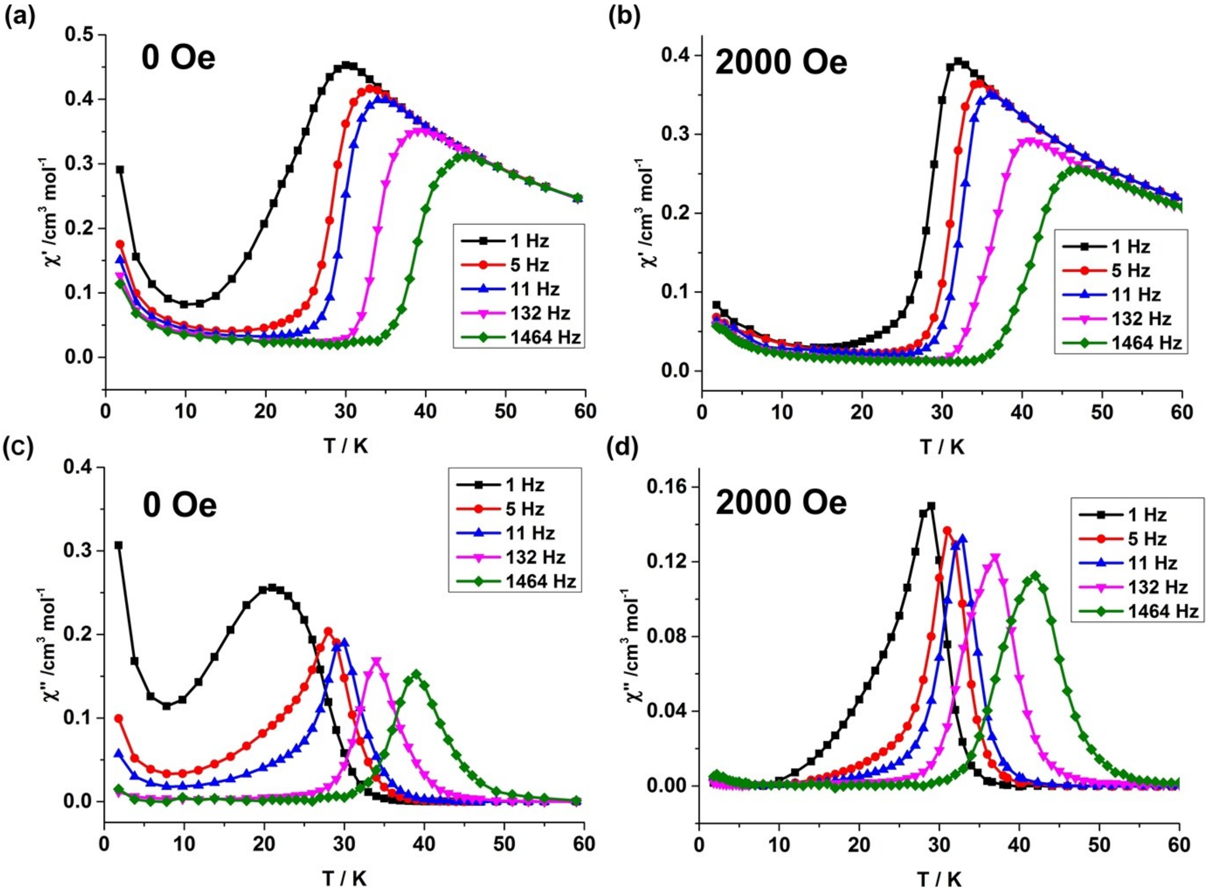

Figure S22. In-phase (χ_M') component (a, b) and out-of-phase (χ_M'') component (c, d) of the ac susceptibility measured in an oscillating ac field of 3.5 Oe under zero applied dc field (a, c) and an applied dc field of 2000 Oe (b, d) for complex **1.**

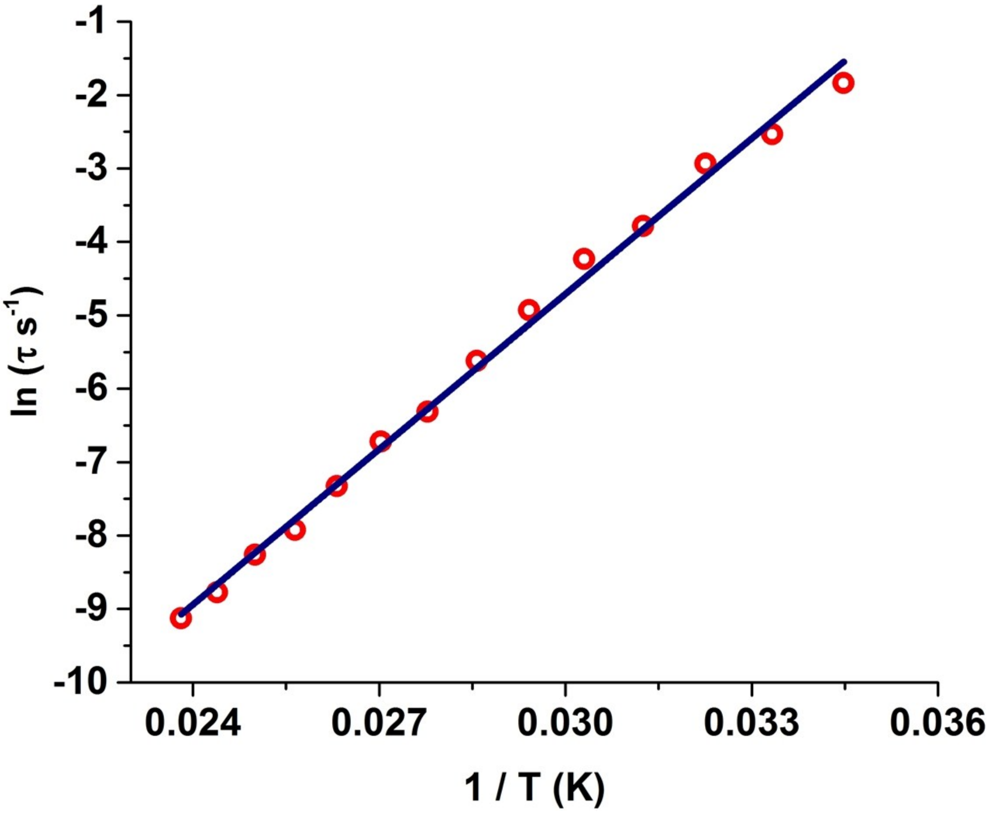

Figure S23. Plot of the relaxation time τ (logarithmic scale) versus T^{-1} for **1** under an applied dc field of 2000 Oe; the solid red line corresponds represents fitting to the Orbach relaxation process (U_{eff} = 705.3 K and a pre-exponential factor (τ_o) = 5.83 x 10^{-12} s).

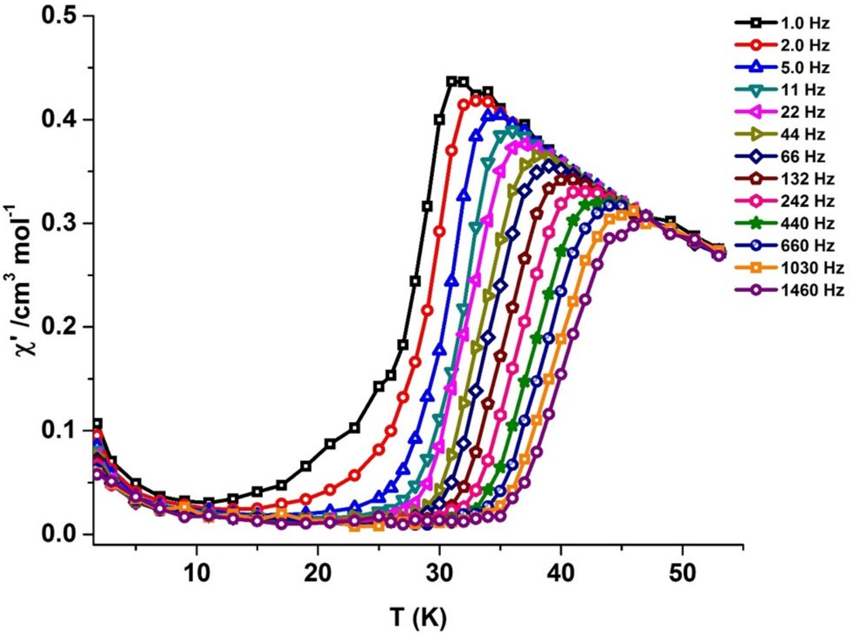

Figure S24. In-phase (χ_M') component of the ac susceptibility measured in an oscillating ac field of 3.5 Oe and zero applied dc field for **1′.**

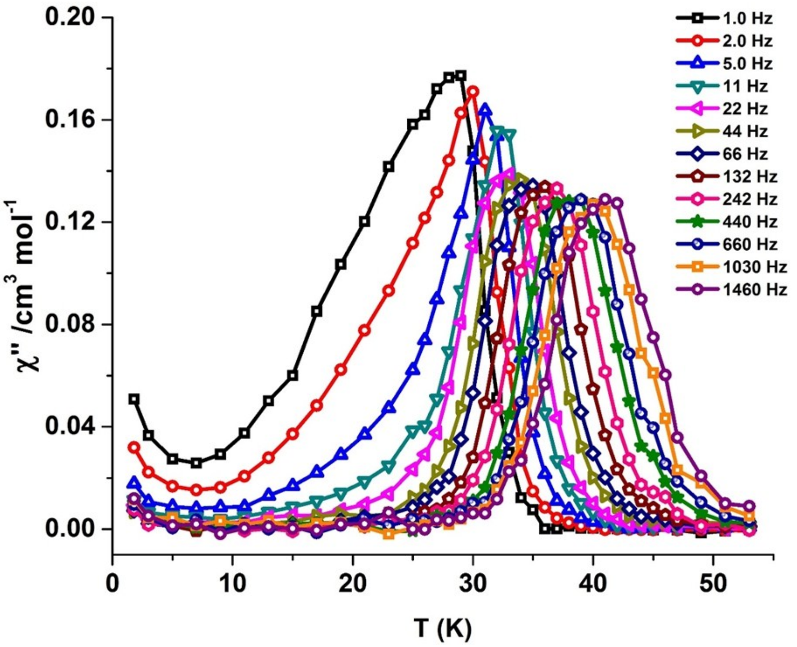

Figure S25. Out-of-phase (χ_M'') component of the ac susceptibility measured in an oscillating ac field of 3.5 Oe and zero applied dc field for **1′.**

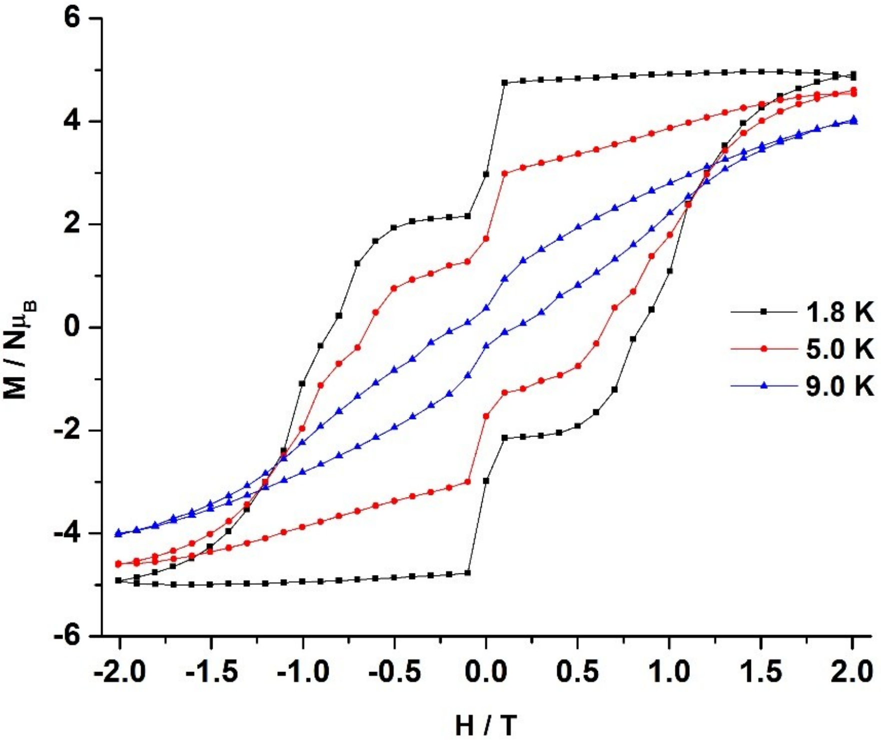

Figure S26. Field dependence of magnetization plot at indicated temperatures for **1′** at an average sweep rate of 0.0018 T s^{-1}.

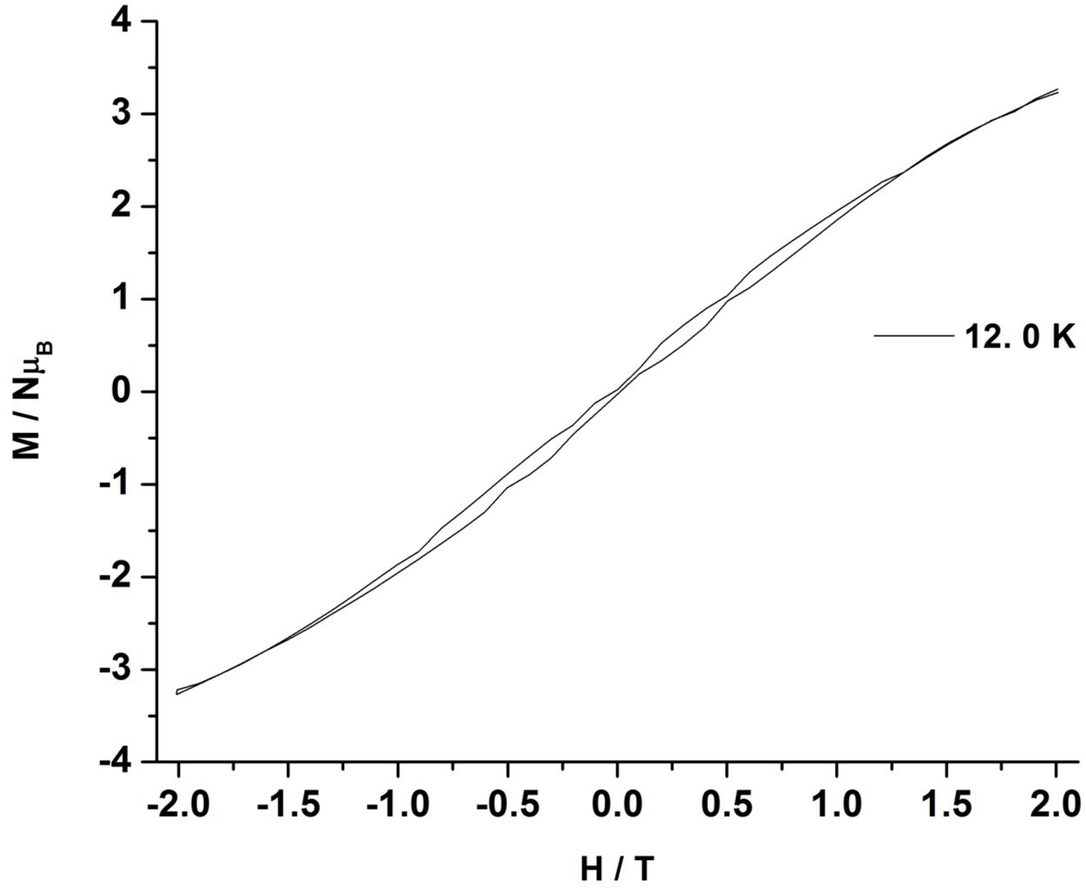

Figure S27. Field dependence of magnetization plot at 12.0 K for **1′** at an average sweep rate of 0.0018 T s^{-1}.

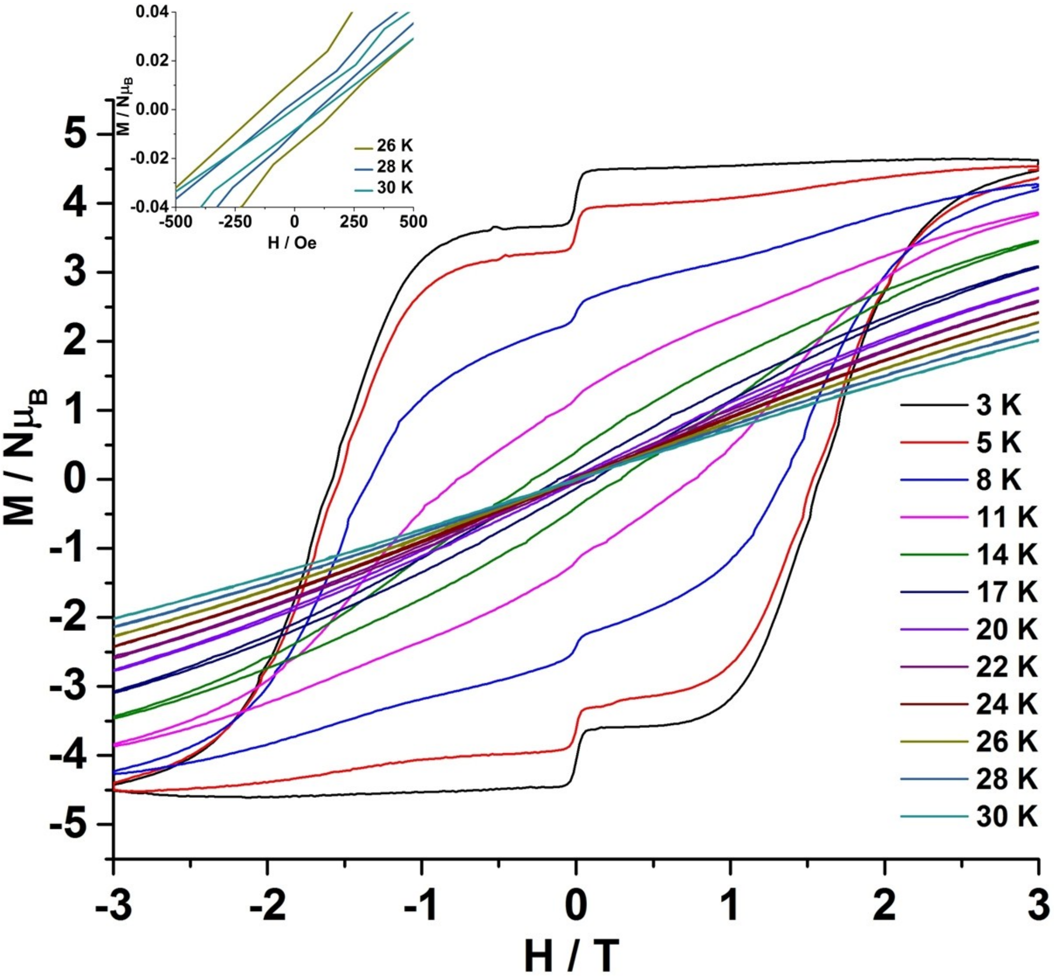

Figure S28. Field dependence of magnetization plot at 12.0 K for **1′** at a continuous sweep rate of 0.02 T s^{-1}.

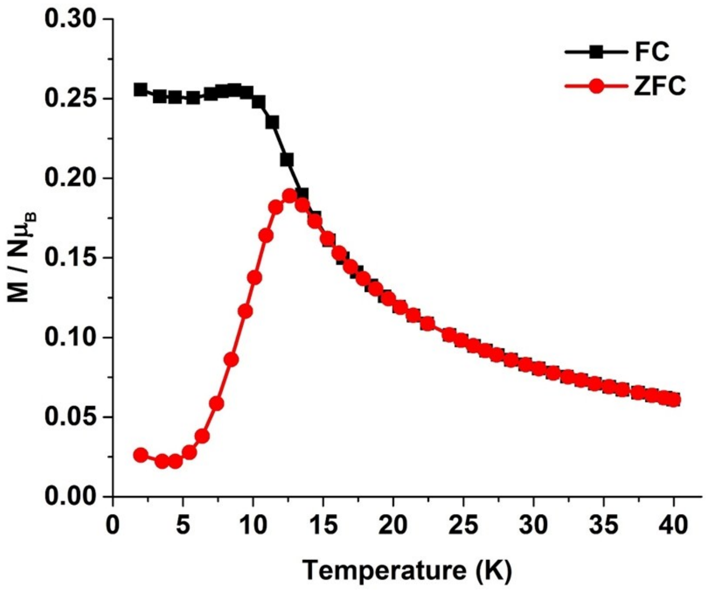

Figure S29. Plot of zero field-cooled (red) and field-cooled (black) magnetization vs temperature for **1′**.

Table S6. SINGLE_ANISO computed energy barrier and angles between the g_{zz} axes of KDs for **1**

Energy (K)	g_{zz}	Angles between g_{zz} axis of KDs
0	19.86	-
461.61	17.08	5.97
688.31	16.53	93.95
758.51	8.22	48.73
774.24	8.51	18.80
897.95	7.67	30.01
924.49	9.61	22.77
1028.37	17.14	52.31

Table S7. Composition of wave functions of the ground J = 15/2 of $Dy^{3+}$for complex **1** as derived from SINGLE_ANISO calculations.

w.f.	m_J	c_i		w.f	m_J	c_i	
		real	*imag.*			*real*	*imag.*
1	-7.5	-0.937649	0.346655	2	-7.5	0.000000	0.000000
	-6.5	-0.000554	0.000395		-6.5	-0.000001	0.000002
	-5.5	-0.001202	0.000675		-5.5	-0.000005	0.000003
	-4.5	0.024627	-0.001114		-4.5	-0.000050	-0.000037
	-3.5	-0.000586	0.001077		-3.5	0.000014	0.000016
	-2.5	-0.001209	-0.001684		-2.5	-0.000024	-0.000004
	-1.5	-0.003401	-0.004012		-1.5	0.000010	-0.000398
	-0.5	0.000055	0.000028		-0.5	-0.000107	0.000148
	0.5	0.000152	0.000102		0.5	0.000042	-0.000046
	1.5	0.000148	0.000370		1.5	0.001799	-0.004942
	2.5	0.000022	-0.000012		2.5	-0.000550	0.001999
	3.5	0.000008	-0.000020		3.5	0.000923	0.000807
	4.5	0.000034	-0.000052		4.5	0.023486	-0.007495
	5.5	-0.000006	-0.000001		5.5	0.001362	0.000217
	6.5	0.000002	0.000001		6.5	-0.000656	-0.000178
	7.5	0.000000	0.000000		7.5	0.999678	0.000000

3	-7.5	-0.000007	0.000022	4	-7.5	0.00026	-0.000845
	-6.5	0.005513	-0.004722		-6.5	-0.344192	0.928896
	-5.5	0.001626	0.000958		-5.5	-0.117172	-0.001148
	-4.5	-0.000096	0.000111		-4.5	-0.004473	-0.014147
	-3.5	-0.000221	0.000036		-3.5	0.035798	-0.044843
	-2.5	0.000013	0.001945		-2.5	0.008683	0.005287
	-1.5	0.002076	-0.002018		-1.5	-0.009705	0.002111
	-0.5	-0.004317	-0.002011		-0.5	-0.033815	-0.002093
	0.5	-0.007927	0.032939		0.5	-0.000655	-0.004717
	1.5	0.004867	-0.008658		1.5	0.002539	-0.001392
	2.5	-0.002505	-0.009853		2.5	0.001856	0.000583
	3.5	-0.053377	0.021054		3.5	-0.0001	0.000201
	4.5	0.012211	0.008429		4.5	0.000134	-0.000059
	5.5	0.033306	-0.112345		5.5	-0.000439	-0.001836
	6.5	-0.989013	0.056286		6.5	-0.006132	0.003884
	7.5	-0.000884	0.000000		7.5	-0.000023	0.000000
5	-7.5	-0.003223	-0.000276	6	-7.5	0.000614	0.000053
	-6.5	-0.013566	-0.043858		-6.5	-0.003257	-0.006309
	-5.5	-0.076816	0.055284		-5.5	0.071452	-0.035303
	-4.5	-0.034976	-0.032662		-4.5	0.00962	0.001067
	-3.5	0.029898	-0.049747		-3.5	0.000422	-0.010233
	-2.5	0.171457	0.00679		-2.5	0.03127	-0.001287
	-1.5	0.099285	0.359644		-1.5	-0.025413	-0.097508
	-0.5	-0.599358	0.369259		-0.5	-0.443362	0.323628
	0.5	-0.414114	-0.360298		0.5	0.565645	0.41908
	1.5	0.033645	-0.094982		1.5	0.129627	-0.349855
	2.5	0.031045	0.003952		2.5	-0.171411	-0.007873
	3.5	0.000453	-0.010231		3.5	0.025542	0.052118
	4.5	0.009676	-0.000242		4.5	0.037636	-0.029557
	5.5	-0.068177	-0.041274		5.5	-0.071816	-0.06164
	6.5	-0.003784	0.006008		6.5	0.01726	-0.04254
	7.5	-0.000616	0.000000		7.5	-0.003234	0.000000
7	-7.5	0.000403	0.000721	8	-7.5	0.004408	0.007885
	-6.5	0.0015	0.016887		-6.5	-0.048256	0.049696
	-5.5	0.19721	-0.087642		-5.5	0.463965	0.232934
	-4.5	0.062264	0.057446		-4.5	-0.006136	0.289361
	-3.5	0.03707	-0.039169		-3.5	-0.154699	0.08147
	-2.5	0.090964	0.03873		-2.5	-0.205642	-0.178735
	-1.5	-0.022067	0.249545		-1.5	0.151681	-0.506669
	-0.5	-0.202537	0.078116		-0.5	-0.283296	-0.021102
	0.5	0.15666	0.23698		0.5	-0.030648	-0.214904
	1.5	-0.368235	0.379637		1.5	-0.20705	0.141032
	2.5	0.256358	0.092279		2.5	0.078194	0.0605
	3.5	-0.004377	-0.174786		3.5	0.0161	-0.051471
	4.5	-0.249578	0.146556		4.5	0.080525	0.026315
	5.5	0.42972	0.291311		5.5	-0.019733	-0.214903
	6.5	-0.01983	0.066371		6.5	0.015472	-0.006931
	7.5	0.009033	0.000000		7.5	-0.000826	0.000000

Table S8. SINGLE_ANISO computed energy barrier and angles between the g_{zz} axes of KDs for **1a**

Energy (K)	g_{zz}	Angles between g_{zz} axis of KDs
0	19.87	-
618.61	17.04	3.51
1082.70	14.38	8.85
1312.66	16.81	95.62
1346.03	10.34	34.78
1384.08	0.91	65.36
1435.41	8.15	33.48
1522.10	16.55	57.42

Table S9. SINGLE_ANISO computed energy barrier and angles between the g_{zz} axes of KDs for **1b**

Energy (K)	g_{zz}	Angles between g_{zz} axis of KDs
0	19.86	-
442.4	17.09	5.99
613.3	16.60	93.86
692.1	7.87	78.31
726.7	13.31	18.7
799.7	7.90	17.9
870	9.99	19.8
977	17.25	51.7

Table S10: DFT computed NPA charges for Dy(III)-SIM

Atom Label	**1**	**1a**	**1b**
Dy1	1.75240	1.75819	1.75733
O1	-1.10596	-1.12163	-1.08936
O2	-1.10632	-1.12851	-1.08988
O5	-0.89851	-0.84423	-0.89954
O6	-0.86015	-0.82226	-0.85703
O7	-0.87836	-0.82825	-0.87939
O8	-0.83298	-0.79109	-0.83419
O9	-0.81752	-0.77249	-0.819

Table S11. SINGLE_ANISO computed energy barrier and angles between the g_{zz} axes of KDs for model complex of **1c** (without water molecules in equatorial positions).

Energy (K)	g_{zz}	Angles between g_{zz} axis of KDs
0	19.90	-
951.33	16.93	0.62
1838.11	14.06	0.43
2544.57	11.41	1.28
2980.55	8.93	7.62
3158.18	3.68	42.77
3207.82	13.31	87.32
3274.10	18.69	96.97

Table S12. SINGLE_ANISO computed energy barrier and angles between the g_{zz} axes of KDs for **2**

Energy (K)	g_{zz}	Angles between g_{zz} axis of KDs
0	13.33	-
19.41	13.34	65.39
53.92	8.51	56.05
93.42	8.18	90.75
106.42	10.64	26.47
127.55	12.04	83.97
157.50	15.87	60.55
579.68	17.92	17.60

Table S13. SINGLE_ANISO computed energy barrier and angles between the g_{zz} axes of KDs for **2a**

Energy (K)	g_{zz}	Angles between g_{zz} axis of KDs
0	17.41	-
41.77	14.56	122.32
67.29	2.64	47.26
78.79	10.55	57.95
84.98	1.14	148.36
117.85	16.58	145.73
244.44	15.56	73.37
797.65	17.92	77.08

Table S14. SINGLE_ANISO computed energy barrier and angles between the g_{zz} axes of KDs for **2b**

Energy (K)	g_{zz}	Angles between g_{zz} axis of KDs
0	13.41	-
25.54	12.97	61.02
62.48	8.84	58.13
97.55	12.48	21.75
107.56	6.38	82.12
138.79	13.42	90.16
168.69	15.75	61.64
557.65	17.91	16.65

Table S15. Composition of wave functions of the ground $J = 15/2$ of $Er^{3+}$for complex **2** as derived from SINGLE_ANISO calculations.

w.f.	m_J	c_i		w.f	m_J	c_i	
		real	*imag*			*real*	*imag*
1	-7.5	0.001244	0.002419	2	-7.5	-0.132756	-0.258199
	-6.5	-0.005166	0.001748		-6.5	0.548492	-0.352884
	-5.5	-0.00138	-0.0025		-5.5	0.206564	0.31818
	-4.5	-0.008183	-0.003121		-4.5	0.363724	-0.36696
	-3.5	-0.031524	-0.002482		-3.5	-0.058229	-0.178312
	-2.5	0.016056	-0.017366		-2.5	-0.135699	0.081773
	-1.5	0.054692	0.0004		-1.5	0.058216	-0.037082
	-0.5	0.018384	0.031336		-0.5	0.086996	-0.029421
	0.5	0.013615	0.090821		0.5	-0.036275	-0.002021
	1.5	0.006358	-0.068729		1.5	0.025364	0.048456
	2.5	0.010673	-0.158073		2.5	0.008102	-0.02222
	3.5	0.185205	-0.02975		3.5	-0.016622	-0.0269
	4.5	-0.160033	0.491268		4.5	0.006517	0.00585
	5.5	-0.377421	-0.038213		5.5	-0.002854	-0.000084
	6.5	-0.063027	0.649152		6.5	0.000808	0.005394
	7.5	0.290328	0.000000		7.5	0.00272	0.000000

3	-7.5	0.004554	-0.002537	4	-7.5	-0.055653	0.030998
	-6.5	0.019723	0.018903		-6.5	-0.112394	-0.140605
	-5.5	-0.073413	0.059327		-5.5	0.255614	-0.165353
	-4.5	-0.066085	-0.054922		-4.5	0.004111	0.127339
	-3.5	-0.198852	0.053368		-3.5	0.322943	-0.35642
	-2.5	0.106907	-0.047484		-2.5	-0.289293	-0.316486
	-1.5	0.267386	-0.122599		-1.5	-0.196089	0.278076
	-0.5	0.070947	0.17914		-0.5	0.344245	0.091514
	0.5	-0.25621	0.247458		0.5	-0.025189	-0.191024
	1.5	-0.306621	-0.147517		1.5	-0.293251	0.023005
	2.5	0.098731	-0.41726		2.5	0.116502	-0.010538
	3.5	0.455565	0.154232		3.5	0.199691	-0.050138
	4.5	0.058372	0.113247		4.5	-0.031009	0.080138
	5.5	0.303771	0.020074		5.5	0.093004	0.016106
	6.5	0.029772	-0.177527		6.5	0.008033	-0.026111
	7.5	-0.063703	0.000000		7.5	-0.005213	0.000000

Table S16: DFT computed NPA charges for Er(III)-complex

Atom Label	**2**	**2a**	**2b**
Er1	1.70306	1.70959	1.70841
O1	-1.09908	-1.1164	-1.08241
O2	-1.09873	-1.12188	-1.08212
O5	-0.81647	-0.777	-0.81777
O6	-0.76213	-0.725	-0.76359
O7	-0.83688	-0.7916	-0.83834
O8	-0.79414	-0.7561	-0.79547
O9	-0.84225	-0.8041	-0.83958

Table S17. SINGLE_ANISO computed crystal field parameters of complex **1**

k	q	B_k^q	k	q	B_k^q
**2**	-2	-0.14966940793717E+00	**4**	-4	-0.16873792506758E-02
	-1	-0.19984864777002E+01		-3	0.53295551280434E-02
	0	-0.28233517290510E+01		-2	0.19864160231553E-02
	1	-0.64664039023571E+00		-1	0.94240020450585E-02
	2	0.26501718888657E+00		0	-0.12332571296602E-01
				1	0.26189545323991E-02
				2	-0.25005224838227E-02
				3	0.20326651840394E-01
				4	0.11596760965258E-02

Table S18. SINGLE_ANISO computed crystal field parameters of complex **2**

k	q	B_k^q	k	q	B_k^q
**2**	-2	-0.10374829093623E-01	**4**	-4	0.21485682384116E-02
	-1	-0.20753682654727E+01		-3	0.31818593677115E-02
	0	0.11330610203096E+01		-2	0.24328370791822E-02
	1	0.15908832573118E+00		-1	-0.42553501878123E-01
	2	0.56402864355649E-01		0	0.40324486716717E-02
				1	-0.28922941277099E-02
				2	-0.13211821835700E-01
				3	-0.61772128377640E-02
				4	0.47178905874170E-03
